# Non-coding Y RNA fragments in a complex with YBX1 modulate PARP1 residency at DNA double strand breaks

**DOI:** 10.1093/nar/gkaf517

**Published:** 2025-06-18

**Authors:** Annabelle Shaw, Kamal Ajit, Manon Chataignier, Monika Gullerova

**Affiliations:** Sir William Dunn School of Pathology, University of Oxford, South Parks Road, OxfordOX1 3RE, United Kingdom; Sir William Dunn School of Pathology, University of Oxford, South Parks Road, OxfordOX1 3RE, United Kingdom; Sir William Dunn School of Pathology, University of Oxford, South Parks Road, OxfordOX1 3RE, United Kingdom; Sir William Dunn School of Pathology, University of Oxford, South Parks Road, OxfordOX1 3RE, United Kingdom

## Abstract

To protect genome integrity from pervasive threats of damage and prevent diseases like cancer, cells employ an integrated network of signalling pathways called the DNA damage response. These pathways involve both protein and RNA components, which can act within the damaged cell or be transferred intercellularly to influence population-wide responses to damage. Here, we show that radioprotection can be conferred by damage-derived exosomes and is dependent on YBX1-packaged Y3-derived ysRNA. In recipient cells, ysRNAs are methylated on cytosine and bound by m5C reader, YBX1. YBX1/ysRNA localise at double strand break (DSB) sites to promote efficient DNA repair and cell survival through complex formation with PARP1. YBX1 modulates PARP1 auto-modification by facilitating ysRNA ADP-ribosylation, promoting increased PARP1 residency at DSBs. Our data highlight an unprecedented role for these under-studied species of small non-coding RNAs, identifying them as a novel substrate for PARP1 mediated ADP-ribosylation with a function in DNA repair.

## Graphical Abstract

**Graphical Abstract:**
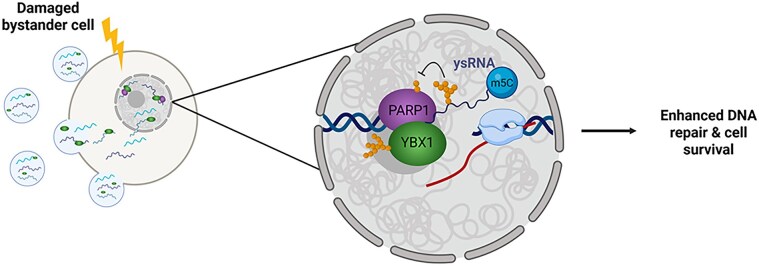

## Introduction

The human genome is under constant threat of damage from endogenous and exogenous sources, resulting in genome instability if improperly repaired. Cells therefore utilise a complex network of signalling pathways, collectively termed the DNA damage response (DDR), to detect and repair these lesions, thus ensuring genome integrity and preventing diseases such as cancer [1, 2]. DNA double strand breaks (DSBs) represent the most cytotoxic lesions which can be induced by ionising radiation (IR) and are repaired via two major pathways: fast, but error-prone, non-homologous end-joining, or high-fidelity homologous recombination [1]. In addition to protein effectors, there is increasing evidence for major roles of RNA, particularly non-coding RNA (ncRNA), in DSB repair. These functions can be classified as in *cis* or in *trans* to DSBs [3].

It recently emerged that DDR can be communicated intercellularly via exosomes, a subset of extracellular vesicles (EVs) with a diameter of 40–150 nm [4]. Many studies have reported alterations to exosome cargo, including protein and RNA, following IR treatment of the cells from which they are secreted [5–9]. Results of such cargo transfer include protection of healthy bystander cells, which are in the vicinity of irradiated cells but are not exposed themselves, against subsequent DNA damaging insults [10, 11]. Such intercellular communication generates a co-operative response to DNA damage, particularly recurring insults, which can increase the overall survival of a cell population [12].

The multifunctional nucleic acid binding protein, Y-Box Binding Protein 1 (YBX1), is capable of binding to various forms of DNA and RNA [13] and has been implicated in packaging of messenger RNA (mRNA), microRNA (miRNA) and other small non-coding RNA (sncRNA) species into EVs [14–17]. Among many other functions, YBX1 is also involved in DNA repair. While the full extent of YBX1 involvement in DNA repair is still under study, it is known to translocate to the nucleus upon genotoxic stress, associate with DNA damage markers such as Ser139-phosphorylated histone H2AX (γH2AX), and participate in DNA repair protein complexes [18–20]. YBX1 therefore represents a strong candidate for involvement in intercellular radioprotection, due to its dual role in RNA packaging and DNA repair.

One example of YBX1-packaged exosomal RNA is Y RNA [16]. Y RNAs represent a class of ncRNA that are transcribed by RNA polymerase III from four genes in the human genome, giving rise to four RNAs from distinct promoters: Y1, Y3, Y4, and Y5. Ranging from 84 to 113 nucleotides (nt) in length, these RNAs form hairpin structures that facilitate their interaction with effector proteins, including Ro60 and La [21–23]. Y RNA functions are overall poorly characterised, although they reportedly include regulation of Ro60 subcellular localisation, RNA processing and quality control, DNA replication and stress responses, including promotion of cell survival following UV irradiation [24–26]. Full length Y RNA may also be processed into shorter fragments of 24–32 nt, termed Y RNA-derived fragments (ysRNAs). This cleavage is thought to be facilitated by RNase L and may occur in a caspase-dependent manner during apoptosis, although ysRNAs could also be detected in non-stressed cells, tumour cells, and cells stressed with poly(I:C) [27–29]. However, the functions of these fragments remain largely unknown.

NSUN2 and DNMT2 are two essential RNA methyltransferases involved in the modification of cytosine to form 5-methylcytosine (m5C) in RNA. NSUN2, a member of the NOP2/Sun RNA Methyltransferase family, plays a crucial role in catalysing this methylation process across various RNA species, including tRNA, mRNA, and non-coding RNAs, while DNMT2 specifically methylates cytosine at the 38th position in tRNA Asp (GTC), forming m5C. The activity of DNMT2 is essential for maintaining tRNA stability and ensuring proper decoding during translation [30]. Additionally, both NSUN2 and DNMT2 play significant roles in stress response and are involved in DDR pathways, highlighting their multifaceted role in cellular protection and repair mechanisms [31]. YBX1 is an m5C reader; it specifically binds to m5C-modified RNA, influencing the fate of these RNAs within the cell. YBX1’s ability to recognize and bind m5C-modified RNA underscores its role in gene expression regulation and the cellular response to DNA damage [32].

In line with its DNA repair role, YBX1 has recently been reported as an interactor and regulator of PARP1 [33, 34]. Primarily known as a DNA single strand break repair factor, PARP1 has recently also been implicated in DSB repair [35, 36]. Following activation, PARP1 can modify both itself and other target proteins by ADP-ribosylation, resulting in a readable signal that is capable of recruiting DNA repair proteins to the lesion, as well as enabling chromatin remodelling events that enhance repair [35–38]. Beyond its role in modifying proteins, PARP1 can also add ADP-ribose polymers to DNA substrates, while other PARP family members have been implicated in RNA modification [39]. Furthermore, PARP1 is able to bind various types of RNA, including mRNA, miRNA, small nucleolar RNA, and lncRNA [40, 41], and has been shown to play a significant role in RNA polyadenylation, export, and stability, as well as ribosome biogenesis [42]. However, the role of PARP1 in modification of RNA at DSBs and its relevance to repair is not known.

Here, we report that exosome-mediated radioprotection is dependent on YBX1-packaged Y3-derived ysRNA which enters the nucleus of bystander cells upon DNA damage. These ysRNAs are methylated by NSUN2 and interact with YBX1 at DSB sites to promote efficient DNA repair and cell survival through complex formation with PARP1. YBX1 facilitates ADP-ribosylation of ysRNA, modulating PARP1 auto-modification and therefore promoting increased PARP1 residency at DSBs to enhance DNA repair.

## Materials and methods

### Cell lines and cell culture

HeLa, HEK293T, YBX1-/- HEK293T (gifted from the Schekman Lab), and PARP1-/- HEK293T (gifted from the Ahel Lab) cells were maintained at 37°C and 4% CO_2_ in DMEM high glucose medium (Life Technologies #31 966 047), supplemented with 10% fetal bovine serum (FBS) (Sigma-Aldrich #F9665), 2 mM L-glutamine (Life Technologies #25 030 024) and 100 units/ml penicillin-streptomycin solution (Life Technologies #15 140 122). TrypLE Express was used to passage cells once 70% confluency reached.

IR was delivered at indicated doses (2, 4, 5, or 10 Gy) by source exposure using GRAVITRON RX 30/55.

### FBS pre-clearing

FBS was filtered through a 0.2 μm filter then aliquoted into 70ml Polycarbonate Bottles for ultracentrifugation. Centrifugation was carried out using Beckman XPN-80 and type 45 Ti rotor at 4°C and 100 000 g for 20 h. Clear fraction was collected, filtered and stored at −20°C.

### Exosome isolation

HEK293T cells were seeded into 10 x 15 cm dishes per condition. After 24 h, cell culture medium was replaced with DMEM + 10% exosome-depleted FBS and cells were exposed to 10 Gy IR, if applicable. After a further 48 h, culture medium was extracted and centrifuged at 300 g for 5 min to pellet any cells, centrifuged at 16 500 g for 20 min to pellet microvesicles, and filtered through a 0.22 μm PES filter (Corning). Media was then concentrated to 15 ml using a 100 kDa MWCO tangential flow membrane (Sartorius Vivaflow 50R), followed by further concentration to 2 ml by centrifugation at 4000 g for ∼15 min using 100 kDa concentrators (Fisher #28–9323-63). For clear medium (CM) control, medium that had not been incubated with cells was treated in the same manner.

Around 2 ml of samples were loaded into AKTA pure system with UV flow cell (GE Healthcare), connected to a Sepharose 4 fast flow gel-filtration column (GE Healthcare, #170 149 001) in DPBS, via a 2 ml loop. UNICORN software was used to set flow rate to 0.5 ml/min with 500 μl fraction collection and no protein collection, and to visualise absorbance at 280 nm. EV fractions were collected based on UV trace and pooled. Further concentration to 200 μl was achieved by centrifugation at 4000 g using 100 kDa centrifugal filters (Merck #UFC210024).

### Nanoparticle tracking analysis

Exosome concentration and size distribution measured using NanoSight NS500 (Malvern) and NanoSight 3.2 software. Concentrated, pooled EVs were diluted 1:500 in PBS. 5 x measurements were recorded for 10 s each.

### Exosome transfer

HEK293T cells were seeded at 20% confluency into 6 cm dishes for western blotting, or onto poly-L-lysine coated glass coverslips for immunofluorescence. After 24 h, exosome-depleted DMEM, combined with NDD EVs, DD EVs or an equal volume of PBS, replaced the culture media of 2 x sets of recipient cells. After a further 24 h, one set of recipient cells was subjected to 10 Gy IR. Cells were harvested for immunoblotting or fixed for immunofluorescence 2 or 6 h post-IR treatment.

### Western blot

Cells were centrifuged at 4°C and 500 g for 5 min, before lysis in 5 x the cell pellet volume of RIPA buffer (Thermo Scientific #89 901), supplemented with 1 x protease inhibitor (Merck # 11 873 580 001) and 1 x phosphatase inhibitor (Roche # 4 906 837 001). Samples were sonicated for 10–30 s and their concentration determined using the Pierce BCA Protein Assay Kit (Thermo Scientific #10 678 484), according to manufacturer’s protocol.

Cell lysates were diluted to equal concentrations with ddH_2_O and Laemmli buffer with β-mercaptoethanol (Alfa Aesar #J61337.AD) added to a final 1 x concentration. Samples were denatured at 95°C for 5 min and standard western blotting carried out using 4–15% Mini-PROTEAN TGX Precast protein gels (BioRad #4 561 083) and nitrocellulose membranes (Perkinelmer #NBA085A001EA). Total protein staining was carried out with Ponceau S (Merck #P7170-1L) for 5 min. Bands were visualised using ECL substrate (Thermo Scientific #32 106) and Amersham ECL Hyperfilm (Cytiva # 28–9068-35) according to manufacturer’s instructions. If applicable, the Super Signal Western Blot Enhancer kit (Thermo Fisher # 46 640) was used according to the manufacturer’s instructions. Blots were quantified using ImageJ.

### Immunofluorescence

Following any necessary treatment, cells were fixed and permeabilised for 10 min each at room temperature with 4% paraformaldehyde in PBS (Alfa Aesar #J61899) and 0.2% Triton X-100 (Sigma #X100-500ML) in PBS (0.2% PBST), respectively. Blocking was carried out using 10% FBS in PBS for 2 h at 4°C. Primary antibodies were diluted in 10% FBS in PBS then incubated with coverslips overnight at 4°C in a humidified chamber. Washes were carried out in 0.1% PBST, before incubation with appropriate secondary antibodies diluted in 0.15% FBS in PBS for 2 h at room temperature in a humidified chamber. Coverslips were mounted using Mounting Medium with DAPI (Abcam #ab104139-20ml). Slides were imaged using Olympus Fluoview FV1200 confocal microscope with a 60 x objective lens, and a numerical aperture of 1:40. Images were quantified using CellProfiler 4.2.1 using a custom pipeline.

### Polymerase chain reaction

Genomic DNA was extracted using Phenol/chloroform/isoamyl alcohol (25:24:1, v/v) (Invitrogen #15 593 031) and precipitated with isopropanol. Semi-nested Polymerase chain reaction (PCR) was performed on genomic DNA to verify YBX1 knockout, as described by Shurtleff *et al.* [15] using the following primer pairs: YBX1-F1 (GGTTGTAGGTCGACTGAATTA) and YBX1-R1 (ACCGATGACCTTCTTGTCC), then YBX1-F2 (CGGCCTAGTTACCATCACA) and YBX1-R1 (ACCGATGACCTTCTTGTCC).

### RNA isolation

Total RNA was isolated from whole cell extracts, nuclear/cytoplasmic fractions or streptavidin beads following RNA immunoprecipitation. Samples were combined with an equal volume of TRIzol LS (Invitrogen # 10 296 028) followed by addition of 1-Bromo-3-chloropropane (Sigma-Aldrich # B9673) according to manufacturer’s instructions. Isopropanol precipitation was carried out by overnight incubation at −20°C, in the presence of 75 mM NH_4_OAc and 2 μl GlycoBlue. RNA pellet was washed sequentially with 100% ethanol, 70% ethanol, and 100% ethanol, before resuspension in DEPC-treated water.

Small RNA from EV samples was also isolated by TRIzol LS extraction. This was followed by ethanol precipitation was using the mirVana miRNA Isolation Kit (Life Technologies #AM1561), following the manufacturer's small RNA enrichment procedure. Elution was carried out twice, in 30 μl DEPC-treated water each time.

### Small RNA sequencing

RNA was isolated from EV samples obtained from WT non-irradiated, WT irradiated and YBX1-/- irradiated cells, and quality controlled. Libraries were constructed using NEBNext® Multiplex Small RNA Library Prep Set (New England BioLabs) and sequenced using NextSeq 500.

### Northern blot

RNA samples were prepared to equal concentrations in 2 x TBE-Urea Sample Buffer (Thermo Scientific # LC6876) and denatured at 98°C for 3 min. Separation was carried out on 12% denaturing (50% g/v urea) polyacrylamide gels in 1 x TBE at 450 V for 2.5 h. RNA was transferred to positively charged nylon membranes (Amersham Hybond-N+ #RPN203B) at 5 V for 1 h in 0.5 x TBE, followed by UV-crosslinking and pre-hybridisation in pre-warmed ULTRAhybOligo buffer (Thermo Fisher #AM8663) at 42°C for at least 1 h. DNA probes against target RNA were radiolabelled with ^32^P-ATP (Perkinelmer #BLU502Z500UC), using T4-polynucleotide kinase (NEB #M0201L), for 2 h at 37°C. Radiolabelled probes were purified using Microspin G-25 columns (Merck #GE27-5325-01) and added to membranes for overnight incubation at 42°C with rotation. 2 x washes in 0.1% SSC buffer were then carried out for 15 min each, before subjection to autoradiography with Amersham Hyperfilm (VWR #28–9068-45).

### siRNA, ASO, plasmid, and synthetic oligonucleotide transfection

siRNA was reverse-transfected using Lipofectamine™ RNAiMax (Invitrogen #13 778 075) according to the manufacturer’s protocol, at a concentration of 60 nM. Reverse transfection of ASOs was carried out in the same way, to a final concentration of 125 nM. Plasmid reverse transfections were carried out using Lipofectamine™ LTX and Plus Reagent (Invitrogen #15 338 100) according to manufacturer’s instructions. Plasmids were transfected at a concentration of 500 ng–1 μg. Unmodified ysRNA oligonucleotides were forward transfected at a concentration of 10 nM using Lipofectamine™ RNAiMax. AlexaFluor488-tagged ysRNA oligonucleotides were reverse transfected using Lipofectamine™ RNAiMax at concentrations of 10 nM.

### RNA immunoprecipitation

Following necessary treatments, cells were pelleted by centrifugation at 500 g and 4°C for 5 min, then lysed in 5 x pellet volume of RIP lysis buffer, supplemented with Ribolock RNase inhibitor (Life Technologies #EO0382) and DNase I (NEB #M0303S). Lysates were incubated at 4°C for 30 min with rotation, then centrifuged at 17 000 g and 4°C for 10 min. Supernatant was kept and 10% removed for input. Alternatively, subcellular fractionation was carried out as described below.

Lysates were pre-cleared with streptavidin magnetic beads (NEB #S1420S) for 2 h at 4°C, then incubated with 1 μg relevant biotinylated RNA in 2 x TENT buffer supplemented with Ribolock, 1 x protease inhibitor and 1x phosphatase inhibitor for 30 min at room temperature. Fresh streptavidin magnetic beads were then added to each sample and pulldown was carried out for 1.5 h at 4°C with rotation. For analysis of immunoprecipitated proteins, beads were washed 4 x in ice-cold 1 x TENT buffer and eluted in 2 x Laemmli buffer by incubation at 95°C for 10 min. For analysis of immunoprecipitated RNA, beads were washed 3 x in ice-cold 1 x TENT buffer then 2 x in ice-cold PBS. Beads were resuspended in 100 μl PBS then eluted by incubation with Trizol LS reagent. RNA isolation was then carried as described previously.

### Subcellular fractionation

Cells were harvested from 15 cm dishes by centrifugation at 500 g and 4°C for 5 min. All buffers were supplemented with 1 x protease and phosphatase inhibitors, and 2 μl/ml Ribolock RNase inhibitor. Cell pellet was lysed in 5 x volume of hypotonic lysis buffer (10 mM HEPES pH 7.9, 60 mM KCl, 1.5 mM MgCl_2_, 1 mM EDTA, 1 mM DTT, 0.075% NP-40) and incubated at 4°C for 10 min with rotation. Nuclei were pelleted by centrifugation at 800 g and 4°C for 5 min, and the supernatant collected as the cytoplasmic fraction. Insoluble nuclear remnants were removed by centrifugation at 13 000 rpm and 4°C for 5 min. The nuclear pellet was washed 5 x in hypotonic lysis buffer without NP-40, lysed in 1 x volume of nuclear lysis buffer (20 mM HEPES pH 7.9, 400 mM NaCl, 1.5 mM MgCl_2_, 0.2 mM EDTA, 1 mM DTT, 5% glycerol) supplemented with RNase inhibitor and diluted with 2 x volume of dilution buffer (20 mM HEPES pH 7.9, 1.6% Triton-X-100, 0.2% sodium deoxycholate). Nuclear lysates were sonicated for 10 s at 10 microns, then centrifuged for 10 min at 13 500 rpm and 4°C. The supernatant was collected as the soluble nuclear fraction.

### Slot blot

Immunoprecipitated RNA samples were diluted in TE buffer and denatured at 95°C for 3 min. Positively charged nylon membranes (Amersham Hybond-N+ #RPN203B) were pre-equilibrated in TE buffer and assembled within Bio-Dot Microfiltration Apparatus (Biorad #1 706 542), as per manufacturer’s instructions. Each slot was washed once in TE buffer, then RNA applied to the membrane and a gentle vacuum applied. Slots were washed in TE buffer then left to dry for 10 min. UV-crosslinking was carried out at 2000 J, followed by blocking in 2% milk in PBS for 1 h at room temperature with shaking. Primary antibodies were diluted in 2% milk in PBS and incubated with membrane overnight at 4°C, with shaking. This was followed by 1 h incubation with appropriate secondary antibodies diluted in 2% milk in PBS at room temperature with shaking. RNA was visualised by autoradiography using ECL substrate and Amersham ECL Hyperfilm according to manufacturer’s instructions. Total RNA staining was then carried out via methylene blue incubation for 5 min at room temperature. Blots were quantified using ImageJ.

### Proximity ligation assay

Cells were treated as appropriate, then fixed and permeabilised for 10 min each at room temperature with 4% paraformaldehyde in PBS (Alfa Aesar #J61899) and 0.2% Triton X-100 (Sigma #X100-500ML) in PBS, respectively. Duolink Proximity Ligation Assay (Sigma Aldrich #DUO92101-1KT) was carried out on fixed cells according to manufacturer’s protocol. Coverslips were mounted using Duolink In Situ Mounting Medium with DAPI (Sigma-Aldrich #DUO82040), and stored protected from light at 4°C. Slides were imaged using Olympus Fluoview Spectral FV1200 confocal microscope with a 60 x or 100 x oil immersion objective lens, and a numerical aperture of 1:40. Quantification was predominantly carried out using CellProfiler and the Speckle Count pipeline. If background measurements were high, ImageJ Find Maxima function was used with a cut-off of 700.

### MTT assay

Transfected HeLa cells were reseeded into 96-well plates at a density of 10 000 cells per well, 24 h post-transfection. 48 h post-transfection, plates were subject to 5 Gy irradiation, if applicable. MTT Cell Proliferation Assay Kit (Abcam #ab211091) was used according to manufacturer’s instructions, with 100% DMSO used instead of MTT solvent. Absorbance at 590 nm was measured at 24, 48, and 72 h post-IR.

### Clonogenic assay

HeLa cells were transfected with ASOs in six-well plates, as described above. 48 h after transfection, cells were treated with 2 Gy IR and left to grow for 7–14 days until visible colonies formed. Plates were then washed twice in PBS, incubated with 0.5% crystal violet (Sigma #C6158-100G) in 20% ethanol for 30 min, rinsed in water and left to dry. Plates were scanned and images quantified using the ColonyArea ImageJ plugin.

### Co-immunoprecipitation

Following transfection with YFP-PARP1 plasmid, cells from 15 cm dishes were washed 3 x in ice cold PBS, scraped in PBS and pelleted by centrifugation at 500 g and 4°C for 5 min. Cell pellets were lysed in 5 x volume of co-IP lysis buffer, supplemented with Pierce Universal Nuclease. Lysates were incubated at 4°C for 30 min with rotation, followed by centrifugation at 17 000 g and 4°C for 10 min. Supernatant was diluted in 1.5 x volume co-IP dilution buffer and 10% removed as input. GFP-Trap beads were washed 3 x and resuspended in cold co-IP dilution buffer. Around 50 μl beads were added to each sample and incubated for 2 h at 4°C with rotation. Supernatant was then removed and beads washed 3 x in cold co-IP dilution buffer, followed by elution in 2 x Laemmli buffer at 95°C for 10 min.

### Laser microirradiation

Following necessary treatments, cells were seeded onto 3.5 cm glass-bottom dishes. Prior to imaging, cells were pre-sensitised for 30 min at 37°C using Hoechst 33 342 (10 μg/ml) in phenol red free media. Cells were imaged using an Olympus SoRa spinning disc confocal microscope and a 405 nm FRAP module in a humidified chamber at 37°C and 5% CO2. Cells expressing fluorescently tagged protein of interest were systematically chosen using a 488 nm laser setting. DNA damage was induced by a 405 nm laser and recruitment of the fluorescently labelled proteins was monitored by live cell imaging. Quantification was carried out using ImageJ, with fluorescence intensities of laser stripes measured and normalised to undamaged regions of the same cell.

### Bioinformatic analysis

#### small RNA-seq data processing

Single end reads obtained from NextSeq 500 were 75 bp long. Illumina universal adapters from these reads were trimmed using Cutadapt (version 4.4) (https://cutadapt.readthedocs.io/en/stable/installation.html) in single end mode and the quality of the resulting fastq files were assessed using FastQC (https://www.bioinformatics.babraham.ac.uk/projects/fastqc/). The trimmed reads were then aligned to GENCODE GRCh38 reference genome using STAR aligner [43]. Custom BED file containing GENCODE GRCh38 annotation with all RNA species except miRNAs were created using custom python script. Read counts across various smallRNA species (snoRNA, snRNA, tRNA, vault RNA, and Y RNA) for each alignment file was calculated using BEDTools [44] multicov function. DESeq2 [45] was then used to perform differential expression analysis on the counts obtained using all three replicates for each condition.

#### tRFs analysis

MINTmap [46] was used to obtain read counts for each annotated tRNA-derived fragment (tRF) from MINTBase [47]. The read counts were then normalised to CPM based on total number of tRFs obtained for each sample. T test was then used to compare enrichment of various tRF species between the conditions (*n* = 3) with significance threshold set at *P* value < 0.05 and |log2Fold Change| > 0.1. Differential enrichment of tRFs was then visualised using EnhancedVolcano (https://bioconductor.org/packages/devel/bioc/vignettes/EnhancedVolcano/inst/doc/EnhancedVolcano.html) R package (www.R-project.org). Fold change of commonly differentially enriched tRFs between various conditions were plotted as a heatmap using Matplotlib python package.

#### miRNA analysis

GRCh38 Co-ordinates for mature miRNA annotations were obtained from miRBase [48]. BEDTools multicov was then used to obtain read counts across mature miRNAs for each of the alignment files resulting from alignment of trimmed reads to GENCODE GRCh38. T test was then used to compare enrichment of various miRNA species between the conditions (*n* = 3) with significance threshold set at *P* value < 0.05 and |log2Fold Change| > 0.1. Differential enrichment of miRNAs was then visualised using EnhancedVolcano (https://bioconductor.org/packages/devel/bioc/vignettes/EnhancedVolcano/inst/doc/EnhancedVolcano.html) R package. Fold change of commonly differentially enriched miRNAs between various conditions were plotted as a heatmap using Matplotlib python package.

### Metagene plots

BigWig files containing RPKM normalised read count per nucleotide position was generated for each alignment file using deepTools bamCoverage [49]. ComputeMatrix operation of deepTools was then performed on the bigWig files to calculate the RPKM coverage across annotated Y RNA species. Matrices were then visualised using plotProfile function of deepTools.

### smallRNA proportion plots

Raw coverage across mature miRNA, tRNAs, snoRNAs, snRNAs, Y RNAs and vault RNA were plotted as a pie chart using Matplotlib python package.

### Fragment size distribution plots

BEDTools getfasta was used to extract all reads mapping to co-ordinates of various Y RNA species in different sRNA sequencing samples. The length of these reads was calculated and plotted as a histogram using a custom python script for each of the samples.

### Data and statistical analysis

All statistical analyses were performed in GraphPad Prism 9.3.1. All error bars represent mean ± SEM unless otherwise stated. Statistical testing was performed using the Student’s *t*-test, one-way ANOVA, two-way ANOVA with Bonferroni’s multiple comparison, Mann–Whitney test for two group non-parametric comparison (for PLA foci analysis), or Kruskal–Wallis test with Dunn’s correction for multiple group comparisons. Significances are listed as *P ≤ 0.05, **P ≤ 0.01, ***P ≤ 0.001, ****P ≤ 0.0001.

### Data availability

Data reported in this paper can be shared by the lead contact upon request.

Exosome Small RNA-seq data have been deposited to GEO and can be accessed under GSE269346 with private token: kzklgeiitlmptkd

Any additional information required to re-analyse the data reported in this work paper is available from the lead contact upon request.

## Results

### Exosomes derived from damaged cells mediate protective YBX1-dependent bystander effect

**Figure 1.**
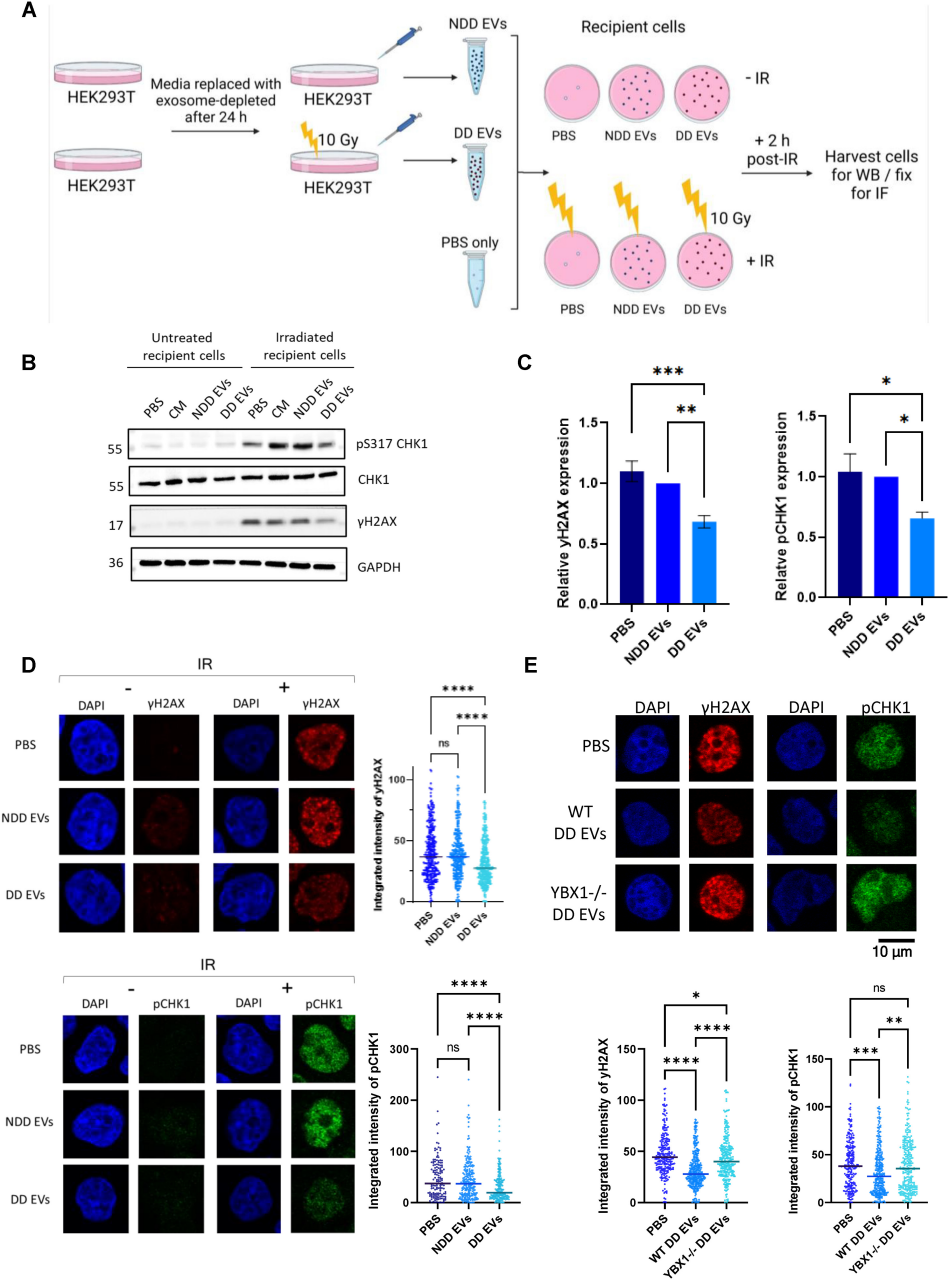
Exosomes derived from damaged cells mediate protective YBX1-dependent bystander effect. (**A**) Schematic overview of exosome transfer experiment, generated in Biorender. NDD EVs = non-damage-derived exosomes, DD EVs = damage-derived exosomes, IR = ionising radiation. (**B**) Representative Western blot showing expression levels of pCHK1 S317 and γH2AX, with CHK1 and GAPDH as loading controls, following exosome transfer (24 h) and irradiation (10 Gy, 2 h) of HEK293T cells. CM = clear media only control. (**C**) Bar chart showing the average fold change in γH2AX and pCHK1 expression levels in IR-treated HEK293T cells upon PBS/exosome transfer. Error bars, mean ± SEM. N ≥ 3, independent experiments. Statistical significance was determined using one-way ANOVA with Tukey’s multiple comparison, *P ≤ 0.05, **P ≤ 0.01, ***P ≤ 0.001. (**D**) Left panel shows representative immunofluorescence images of γH2AX and pCHK1 expression levels upon transfer of NDD EVs, DD EVs, or a PBS control (24 h) in IR-treated (10 Gy, 2 h) and untreated HEK293T cells. Right panel shows quantification from independent experiments where N ≥ 100. Statistical significance was determined using Kruskall-Wallis test with Dunn’s multiple comparison., ****P ≤ 0.0001. (**E**) Top panel shows representative immunofluorescence images of γH2AX and pCHK1 expression levels upon transfer of DD EVs (24 h) from wild type (WT) or YBX1 knockout (YBX1-/-) donor HEK293T cells in IR-treated (10 Gy, 2 h) recipient HEK293T cells. Bottom panel shows quantification from independent experiments where N ≥ 100. Statistical significance was determined using Kruskall-Wallis test with Dunn’s multiple comparison., ****P ≤ 0.0001.

Previous studies in human cells have shown a radioprotective bystander phenotype that is transmitted via exosomes, resulting in increased recipient cell proliferation and survival upon radiation insult when treated with exosomes derived from damaged donor cells [10, 11]. We sought to determine whether this phenotype was mediated by alterations in the cellular response to DNA damage, using exosome transfer experiments (Fig. 1A). Exosomes were isolated by size exclusion chromatography (Supplementary Fig. S1A-F) from the culture medium of HEK293T cells, which were either untreated or treated with 10 Gy IR, giving rise to exosomes that were designated as non-damage-derived (NDD EVs) or damage-derived (DD EVs), respectively. PBS and CM, which had not been incubated with cells, were used as controls. Quality control data showed that pooled EV samples that were used contained exosomal markers, as well as particles in the expected 40–150 nm size range, and were lacking in cellular markers (Supplementary Fig. S1E and F). Recipient HEK293T cells were incubated with these exosomes for 24 h then treated with 10 Gy IR or left untreated, and the expression of DDR markers was analysed. These included: phosphorylation of SMC1, which is a member of the structural maintenance of chromosomes family and can be phosphorylated by ATM; phosphorylation of CHK1, which corresponds to its activation in response to DNA damage and leads to cell cycle arrest; phosphorylation of H2AX, which is a marker for DSBs; and H3K9 methylation which is linked to heterochromatin formation. In addition to being the earliest time point tested at which DDR-related effects become visible (Supplementary Fig. S2), 24 h is the common incubation period employed by other groups for similar exosome transfer experiments and therefore was selected for further studies [6, 10]. Incubation with DD EVs resulted in significantly lower γH2AX and pCHK1 levels in irradiated recipient cells than in those receiving NDD EVs (Fig. 1B-E). This reduction in DNA damage markers was also sustained at later time points (Supplementary Fig. S3A and B) suggesting DD EVs lead to radio-resistance via enhanced DNA repair. It has previously been suggested that RNA species can contribute to bystander effects, including enhancement of radio-resistance [12]. Additionally, a similar radioprotective phenotype was shown to be abrogated by RNase A treatment of exosomes [10], suggesting RNA involvement. We therefore hypothesised that YBX1 functions within this process via its ability to specifically load sncRNA species into exosomes. To test this hypothesis, we utilised a YBX1 knockout cell line (YBX1-/-), which was first validated by Western blot and PCR to show loss of expression at the protein level and an expected genomic deletion surrounding the CRISPR-Cas9 cut site, respectively (Supplementary Fig. S3C and D). Using these knockout cells, we were able to show that the radioprotective phenotype observed in Fig. 1D was diminished when recipient cells were treated with DD EVs from YBX1-/- donor cells (Fig. 1E). These data suggest that DNA damage-derived exosomes confer YBX1-dependent radioprotection to bystander cells via enhanced DNA repair.

### YRNA derived fragments in exosomes are responsive to DNA damage and YBX1 KO

**Figure 2.**
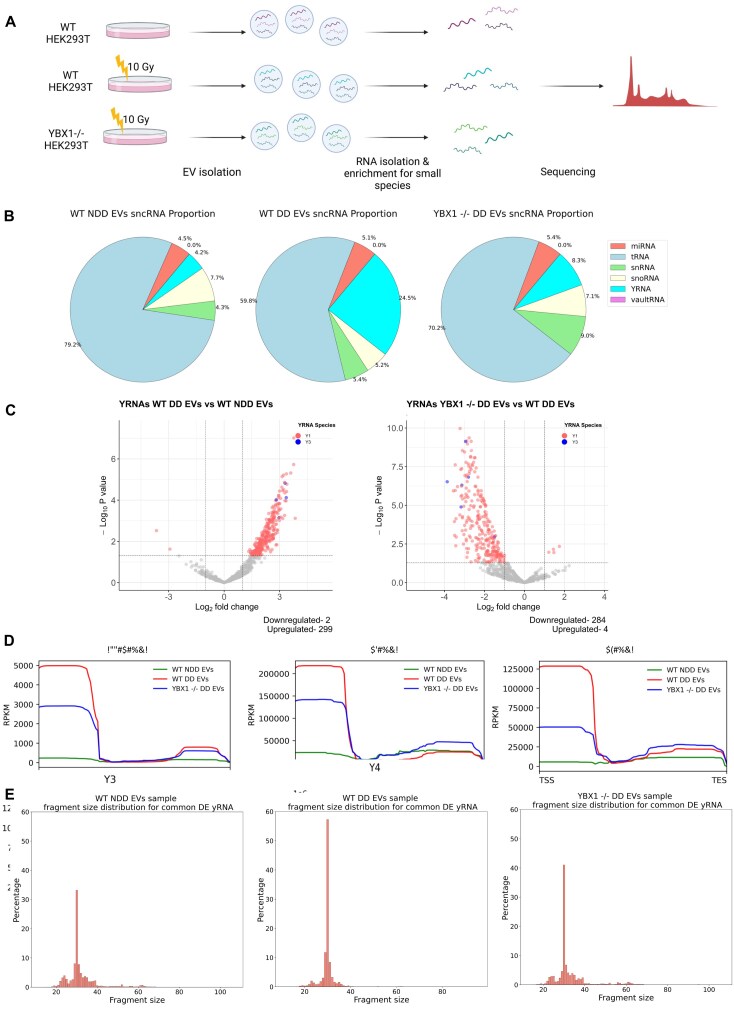
Y RNA derived fragments in exosomes are responsive to DNA damage and YBX1 knockout. (**A**) Schematic overview of method followed to obtain RNA from exosomes for small RNA sequencing, generated in Biorender. (**B**) Pie charts showing the proportion of reads corresponding to different classes of sncRNA obtained from wild-type non-damage-derived exosomes (WT NDD EVs), wild-type damage-derived exosomes (WT DD EVs) and YBX1 knockout damage-derived exosomes (YBX1-/- DD EVs). (**C**) Volcano plots showing differentially expressed Y RNA in exosomes upon damage (left) and YBX1 knockout (right). Coloured points show up- and downregulated Y RNA-corresponding reads with log2FC > 1 and log2FC < −1 and P-adj < 0.001, respectively. Y1 and Y3 associated transcripts are shown in red and blue, respectively. (**D**) Metagene distribution of commonly differentially expressed Y RNAs, Y1 RNA, and Y3 RNA reads mapped along normalised gene length, in relation to transcription start and end sites (TSS and TES, respectively). (**E**) Size distribution of reads corresponding to Y RNA from WT NDD EVs, WT DD EVs, and YBX1-/- DD EVs (left to right, respectively).

Next, we aimed to identify and characterise candidate RNA species which were responsive to both damage and YBX1 knockout conditions and may contribute to the observed phenotype. To do this, we isolated exosomal RNA from wild type (WT) NDD EVs, WT DD EVs, and YBX1-/- DD EVs and performed small RNA sequencing (Fig. 2A). Among the identified species of interest were various tRFs and miRNAs (Supplementary Figs S4 and S5), as well as Y RNA-derived fragments (Fig. 2B and C). Interestingly, Gene Set Enrichment analysis of the common differentially expressed miRNA species (those that were upregulated upon DNA damage in a YBX1-dependent manner) identified pathways including UV response and G2/M checkpoint among targets (Supplementary Fig. S5D). Similar analysis of the common differentially expressed tRF species using the tsRFun database included cell cycle and apoptosis pathways being affected (Supplementary Fig. S4D), both pointing towards possible relevance to radioprotection.

While a large proportion of the reads in our dataset correspond to tRNA fragments (Fig. 2B), reads corresponding to Y RNA-derived fragments appeared more highly and specifically responsive to both damage and YBX1 knockout conditions, particularly those associated with Y1 and Y3 genes (Fig. 2B and C). We therefore decided to focus on Y RNA fragments for further investigation, due to their strong upregulation upon damage in a YBX1-dependent manner. Metagene analysis showed that Y RNA fragments whose presence in exosomes was upregulated upon DNA damage and downregulated upon YBX1 knockout mapped primarily to a region near the transcription start site (TSS) of Y1 and Y3 genes, suggesting these fragments are largely derived from the 5′ end of Y1 and Y3 RNA (Fig. 2D). The most enriched fragment length was 30 nt, corresponding to the previously reported ysRNA size of 24–32 nt [27] (Fig. 2E and Supplementary Fig. S6). We also show via Northern blot that the cellular levels of Y1 and Y3 RNA do not change in response to IR treatment or YBX1 loss (Supplementary Fig. S7). This supports the involvement of YBX1 in a packaging capacity, as opposed to influencing Y RNA biogenesis or processing within exosome donor cells. Overall, these results identified 30 nt 5′ fragments of Y1 and Y3 RNA as candidates for driving the observed radioprotective phenotype.

### Methylated ysRNAs are nuclear and interact with YBX1 at DSBs

**Figure 3.**
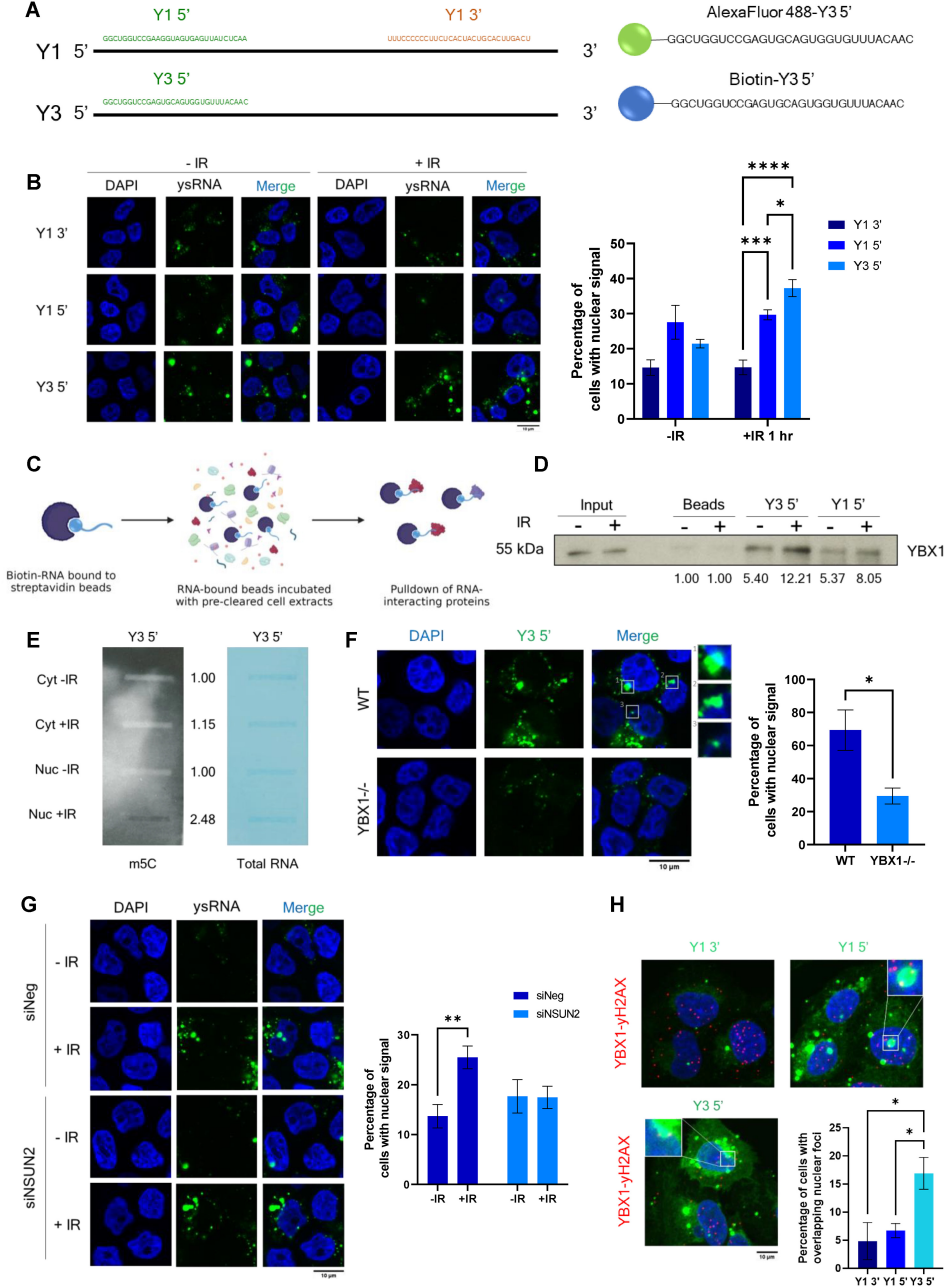
YsRNAs are nuclear, modified by NSUN2 and interact with YBX1 at DSBs. (**A**) Representation of synthetic ysRNA oligonucleotide sequences used for subsequent experiments. (**B**) Representative images (left) and quantification (right) of nuclear localisation of AlexaFluor488-labelled ysRNA oligonucleotides following transfection into HEK293T cells and IR-treatment (10 Gy, 1 h). Mean ± SEM. Quantification was carried out using ImageJ and represented as the percentage of cells per frame with nuclear fluorescent signal (N ≥ 6 frames, at least 70 cells per condition). Statistical significance was determined using two-way ANOVA, *P ≤ 0.05, ***P ≤ 0.001. (**C**) Schematic overview of biotinylated RNA immunoprecipitation, generated in Biorender. (**D**) Co-immunoprecipitation of YBX1 with biotinylated Y3 5′ or Y1 5′ ysRNA oligonucleotide, or beads only control (no RNA) from HEK293T cell nuclear lysates. Numbers represent fold change in band intensity compared with beads only control, normalised to input. Quantified using ImageJ. (**E**) Slot blot for m5C modification (left) and total RNA stain (right) following pulldown of biotinylated Y3 5′ ysRNA oligonucleotide after incubation with cytoplasmic (Cyt) and nuclear (Nuc) fractions from IR-treated (10 Gy, 10 min, +IR) and untreated (-IR) cells. Numbers represent signal intensity of m5C in Y3 5′ pulldown, normalised to corresponding total RNA signal. Quantified using ImageJ. (**F**) Representative images (left) and quantification (right) of nuclear localisation of AlexaFluor488-labelled Y3 5′ oligonucleotides following transfection into wild type (WT) or YBX1 knockout (YBX1-/-) HEK293T cells and IR-treatment (10 Gy, 1 h). Mean ± SEM. Quantification was carried out using ImageJ and represented as the percentage of cells per frame with nuclear fluorescent signal (N ≥ 3, at least 40 cells analysed). Statistical significance was determined using Student’s t-test, *P ≤ 0.05. (**G**) Representative images (left) and quantification (right) of nuclear localisation of AlexaFluor488-labelled Y3 5′ oligonucleotides following transfection into HEK293T cells treated with negative control siRNA (siNeg) or siRNA against NSUN2 (siNSUN2) (60 nM, 48 h) and IR-treatment (10 Gy, 1 h). Mean ± SEM. Quantification was carried out using ImageJ and represented as the percentage of cells per frame with nuclear fluorescent signal (N ≥ 6 frames, at least 80 cells analysed). Statistical significance was determined using one-way ANOVA with Tukey’s multiple comparison, **P ≤ 0.01. (**H**) Representative images (top and bottom left) and quantification (bottom right) of PLA between YBX1 and γH2AX, combined with transfection of AlexaFluor488-labelled synthetic ysRNA oligonucleotides (Y1 3′, Y1 5′, and Y3 5′, 24 h) in U2OS cells. Quantification represented as percentage of cells with overlapping green and red foci per frame, N ≥ 6 frames. Statistical significance determined by Kruskall–Wallis test with Dunn’s multiple comparison. *P ≤ 0.05.

The 30 nt sequences derived directly from the 5′ end of Y1 and Y3 genes accounted for 40% and 34% of total Y RNA reads, respectively. We therefore generated synthetic oligonucleotides corresponding to these sequences for further investigation, termed Y1 5′ and Y3 5′, alongside a control 30 nt sequence derived from the 3′ terminus of the Y1 gene (which was not significantly changing in our dataset), termed Y1 3′. In addition to unmodified oligonucleotides, we also generated fluorescently labelled and biotinylated versions of these oligonucleotides, all of which are represented in Fig. 3A. First, we sought to determine the subcellular localisation of these ysRNAs upon IR treatment in order to assess the likelihood of them influencing DDR. By tracking fluorescently labelled Y1 3′, Y1 5′, and Y3 5′ oligonucleotides in HEK293T cells via microscopy, we observed significantly higher nuclear localisation of Y3 5′ and, to a lesser extent, Y1 5′ upon IR treatment compared with the control Y1 3′ (Fig. 3B and Supplementary Fig. S8A). The same IR-dependent increase in Y3 5′ nuclear localisation was also observed in U2OS cells (Supplementary Fig. S8B).

Next, we aimed to identify associated protein effectors which may help stabilise ysRNA in the nucleus by forming RNPs. YBX1 had previously been identified as an interactor of full length Y3 RNA [50], so we investigated whether YBX1 could also bind 5′ ysRNA in the nucleus of recipient cells. Using synthetic biotinylated ysRNA oligonucleotides, we performed RNA co-immunoprecipitation using both whole cell lysates and nuclear extracts (Fig. 3C) and indeed observe an interaction between Y3 5′ ysRNA and YBX1 which is enhanced upon DNA damage induction (Fig. 3D and Supplementary Fig. S8C). As it has previously been shown that YBX1 is a reader of methylation marks, such as NSUN2-deposited m5C [51], we performed a similar pulldown experiment to determine whether Y3 5′ ysRNA could be m5C-modified. Cytoplasmic and nuclear cell fractions were obtained from either untreated or IR-treated cells (Supplementary Fig. S8D), incubated with biotinylated Y3 5′ ysRNA, and followed by streptavidin pulldown (as depicted in Fig. 3C). However, to check for modification, RNA was eluted from beads instead of protein and analysed by slot blot. Interestingly, we observe that only nuclear fractions from IR-treated cells were able to facilitate m5C modification of Y3 5′ ysRNA, but not cytoplasmic or non-treated nuclear fractions (Fig. 3E and Supplementary Fig. S8E). We then performed the same experiment using nuclear fractions from IR-treated cells which had been depleted of NSUN2 or DNMT2 by siRNA (Supplementary Fig. S8F and G) and show that this methylation is dependent on NSUN2. Furthermore, we hypothesised that the interaction between YBX1 and Y3 5′ ysRNA may be important for maintaining the stability of ysRNA in the nucleus. Indeed, loss of YBX1 results in significant reductions in the level of nuclear ysRNA upon IR treatment compared with wild type (Fig. 3F). As there also appears to be reduced ysRNA signal outside of the nucleus in YBX1-/- cells, we cannot rule out the possibility that YBX1 binding also contributes to the general stability of these species. To investigate whether nuclear NSUN2-mediated methylation of ysRNA could also impact the nuclear stability of ysRNA, we performed a similar immunofluorescence experiment utilising siRNA-mediated knockdown of NSUN2. In control conditions, significantly higher levels of Y3 5′ ysRNA are detected in the nucleus following IR treatment, however the same is not seen upon NSUN2 knockdown (Fig. 3G and Supplementary Fig. S8H). This suggests that NSUN2-mediated methylation, alongside YBX1 binding, can mediate the IR-dependent increase in nuclear ysRNA localisation.

To further investigate whether the YBX1–ysRNA complex has a role in DNA repair, we wanted to determine whether the complex could localise to DSBs. First, we employed a proximity ligation assay (PLA) using antibodies against YBX1 and DSB marker, γH2AX, following IR treatment. This quantitative assay allows for detection of *in vivo* protein–protein interactions [52]. We observed significantly increased numbers of nuclear PLA foci upon IR treatment (Supplementary Fig. S8I), demonstrating an interaction between YBX1 and γH2AX and suggesting that YBX1 localises to DSBs. We then combined PLA with transfection of fluorescently labelled ysRNA oligonucleotides (Fig. 3A) to determine whether the YBX1–ysRNA complex also localised to DSBs. Indeed, we see a significantly higher overlap between Y3 5′ foci and YBX1-γH2AX PLA foci compared with Y1 5′ and Y1 3′ ysRNA (Fig. 3H), suggesting Y3 5′ ysRNA in complex with YBX1 is localised at DSBs. Overall, we show that Y3 5′ ysRNA translocates to the nucleus upon IR, where it becomes methylated by NSUN2 and interacts with YBX1 at DSB sites.

### YsRNA/YBX1 promote efficient DDR and cell survival

**Figure 4.**
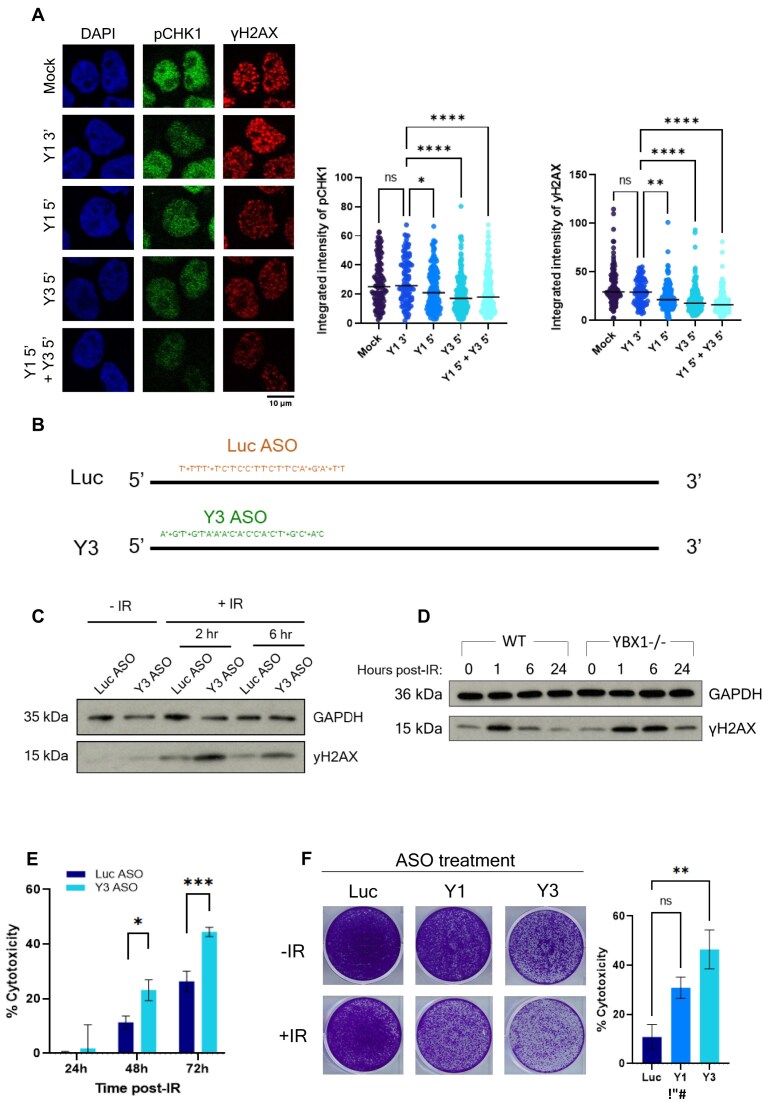
YsRNA/YBX1 promote efficient DDR and cell survival. (**A**) Representative immunofluorescence images (left) and quantification (right) showing levels of pCHK1 S317 and γH2AX following transfection with synthetic ysRNA oligonucleotides, or mock control transfection, and IR-treatment (10 Gy) of HEK293T cells. Nuclear signal intensity was quantified using CellProfiler. N ≥ 100. Statistical significance determined by Kruskal–Wallis test with Dunn’s multiple comparison. *P ≤ 0.05, **P ≤ 0.01, ****P ≤ 0.0001. (**B**) Representation of ASO sequences used to downregulate expression of Y3 RNA (Y3 ASO) and luciferase control (Luc ASO). (**C**) Western blot showing expression of γH2AX and GAPDH as a loading control following IR treatment (10 Gy, +IR, indicated time points) of HEK293T cells treated for 48 h with control (Luc ASO) or Y3-RNA targeting ASO (Y3 ASO). (**D**) Western blot showing expression of γH2AX, and GAPDH as a loading control, in untreated (0) or IR-treated (10 Gy) wild type (WT) or YBX1 knockout (YBX1-/-) HEK293T cells at various time points after damage (1, 6, and 24 h). (**E**) Quantification of MTT assay, showing the cytotoxicity induced by IR (5 Gy) in HeLa cells treated with Y3 or control (Luc) ASO. Mean ± SEM, N ≥ 12. Statistical significance determined using multiple unpaired t-tests. *P ≤ 0.05, ***P ≤ 0.001. (**F**) Representative images (left) and quantification (right) of clonogenic survival assay. HeLa cells treated with control (Luc), Y1 or Y3 ASO (48 h) were untreated (-IR) or subjected to IR (2 Gy, +IR) and growth measured after 5 days. Quantification represented as cytotoxicity upon IR treatment. Mean ± SEM. Statistical significance determined by Kruskal–Wallis test with Dunn’s multiple comparison, **P ≤ 0.01.

In order to confirm that the YBX1–ysRNA complex was physiologically relevant to DSB repair, we interrogated its influence on DDR and cell survival. Using the synthetic ysRNA oligonucleotides described in Fig. 3A, we were able to recapitulate the radioprotective phenotype observed by exosome transfer in Fig. 1D. Briefly, cells were transfected with relevant unmodified oligonucleotides, incubated for 24 h then treated with IR and levels of pCHK1 and γH2AX measured by immunofluorescence. We observe that treating cells with Y3 5′ ysRNA, or a combination of Y3 5′ and Y1 5′ ysRNA, was able to significantly reduce pCHK1 and γH2AX levels (Fig. 4A and Supplementary Fig. S9A), suggesting ysRNA does play a role in enhancing DNA repair. Conversely, when we employ antisense oligonucleotides (ASOs) against Y3 RNA to downregulate its expression (Fig. 4B and Supplementary Fig. S9B), we observe increased levels of γH2AX after IR treatment compared with a luciferase-targeting control ASO (Fig. 4C). Together, these data support Y3 5′ ysRNA involvement in DDR following IR exposure. Interestingly, we observe the same trend in γH2AX phosphorylation when damage is induced in YBX1-/- cells (Fig. 4D), suggesting complementarity in the functions of these two factors.

To ascertain whether this influence on DDR impacted overall cell survival, we performed MTT and clonogenic survival assays in HeLa cells treated with Y3-targeting ASOs (alongside luciferase-targeting and Y1-targeting control ASOs) upon IR treatment. Although there was small reduction in cell survival observed upon Y3 perturbation in untreated cells, relative survival upon IR treatment of Y3 ASO-treated cells (represented as percentage cytotoxicity) was significantly reduced compared with controls (Fig. 4E and F). Together, these data suggest that ysRNA, in complex with YBX1, promotes efficient DNA repair and increased cell survival following IR-induced damage.

### YBX1 interacts with PARP1, facilitating ADP-ribosylation of ysRNA

**Figure 5.**
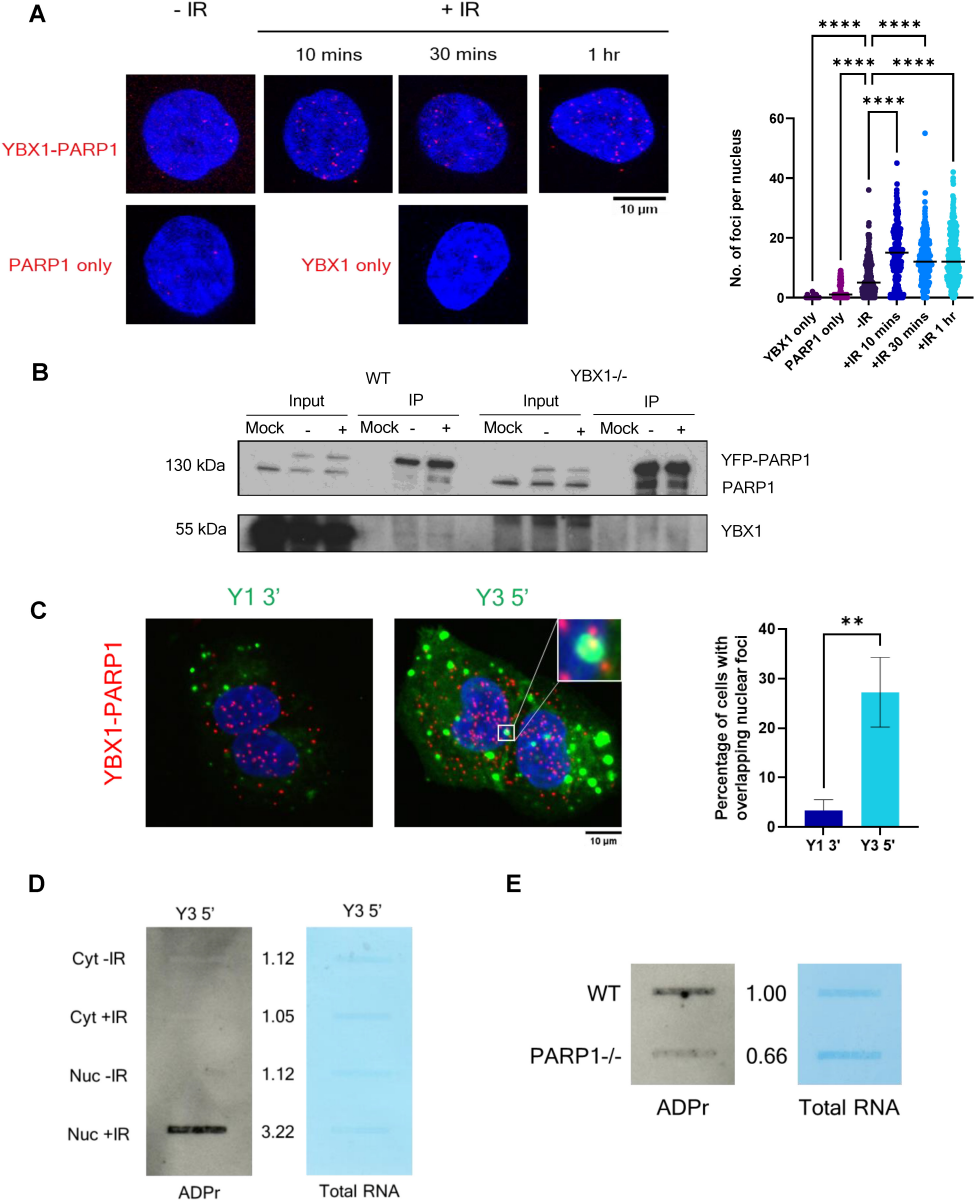
YBX1 interacts with PARP1, facilitating ADP-ribosylation of ysRNA. (**A**) PLA of YBX1 and γH2AX in HeLa cells, with and without IR treatment (10 Gy, +IR, and -IR, respectively), including single antibody control assays. Left panel shows representative images, and right panel shows quantification carried out in CellProfiler. Statistical significance was determined using Kruskall–Wallis test with Dunn’s multiple comparison, ****P ≤ 0.0001. (**B**) Detection of YBX1 in co-immunoprecipitation of YFP-PARP1 from mock-transfected or YFP-PARP1-transfected wild type (WT) and YBX1 knockout (YBX1-/-) HEK293T cells, following subjection to 10 Gy IR (+) or left untreated (-). (**C**) Representative images (left) and quantification (right) of PLA between YBX1 and PARP1 upon IR treatment (10 Gy, 10 min) of U2OS cells, combined with transfection of AlexaFluor488-labelled synthetic ysRNA oligonucleotides (Y1 3′ and Y3 5′). Quantification represented as percentage of cells with overlapping green and red foci per frame, N ≥ 6 frames. Statistical significance determined by Mann–Whitney test. **P ≤ 0.01. (**D**) Slot blot for ADP-ribosylation (ADPr) modification (left) and total RNA stain (right) following pulldown of biotinylated Y3 5′ ysRNA oligonucleotide or beads only control after incubation with cytoplasmic (Cyt) and nuclear (Nuc) fractions from IR-treated (10 Gy, 10 min, +IR) and untreated (-IR) HEK293T cells. Numbers represent signal intensity of ADPr in Y3 5′ pulldown, normalised to corresponding total RNA signal. Quantified using ImageJ. (**E**) Slot blot for ADPr modification (left) and total RNA stain (right) following pulldown of biotinylated Y3 5′ ysRNA oligonucleotide, after incubation with nuclear fractions from IR-treated (10 Gy, 10 min, +IR) wild-type (WT) or PARP1 knockout (PARP1-/-) HEK293T cells. Numbers represent signal intensity of ADPr in Y3 5′ pulldown, normalised to corresponding total RNA signal. Quantified using ImageJ.

Our next goal was to determine how the YBX1–ysRNA complex influences DDR. As little is known about the function of ysRNA, we decided to focus on known roles of YBX1 in DDR. As previously mentioned, YBX1 has been reported to interact with and promote the activity of PARP1 at DSBs [33, 34]. We therefore began by confirming this interaction within cells following IR treatment via PLA and co-immunoprecipitation. Indeed, we show increased PLA foci numbers corresponding to YBX1–PARP1 interaction following IR treatment (Fig. 5A), which was confirmed by co-immunoprecipitation of overexpressed YFP-tagged PARP1 with YBX1 (Fig. 5B). Subsequently, we again combined PLA with fluorescently labelled ysRNA transfection to determine whether ysRNA–YBX1 complexes may also co-localise with PARP1. As hypothesised, we see significantly higher overlap between fluorescently labelled Y3 5′ ysRNA and YBX1-PARP1 PLA foci in the nucleus than control Y1 3′ ysRNA, suggesting formation of a nuclear ysRNA–YBX1–PARP1 complex (Fig. 5C).

YBX1 has previously been reported to become ADP-ribosylated by PARP1 and been suggested to influence its activity through this modification [33]. Furthermore, PARP1 has been shown to bind to RNA substrates through its WGR domain *in vitro* [53]. Therefore, we hypothesised that ysRNA within a complex with YBX1 and PARP1 may also become modified. To test this hypothesis, we performed the same subcellular fractionation and ysRNA pulldown experiment described previously (in relation to Fig. 3E), this time probing for ADP-ribosylation via slot blot. In line with previous results, we observe a band corresponding to ADP-ribosylated Y3 5′ ysRNA, only in nuclear fractions of IR-treated cells, which is significantly higher than that of a control Y1 3′ ysRNA (Fig. 5D and Supplementary Fig. S10A). Furthermore, this experiment was repeated using nuclear fractions of NSUN2 or DNMT2 knockdown cells to determine whether methylation is required for ADP-ribosylation, possibly via the enhancement of ysRNA nuclear stability. Indeed, we observed a reduction in ADP-ribosylation signal upon NSUN2 knockdown (Supplementary Fig. S10B), suggesting methylation is important for further modification of ysRNA. Finally, we observed a reduction in ADP-ribosylation signal when Y3 5′ ysRNA pulldown was carried out using nuclear fractions from IR-treated PARP1 knockout cells (Fig. 5E), suggesting PARP1 is required for ADP-ribosylation of Y3 5′ ysRNA. Together, these data suggest that Y3 5′ ysRNA bound to YBX1 at DSBs is capable of being modified by PARP1 and this is influenced by NSUN2 activity.

### YBX1/ysRNA promote PARP1 residency at DSBs by affecting its auto-modification

**Figure 6.**
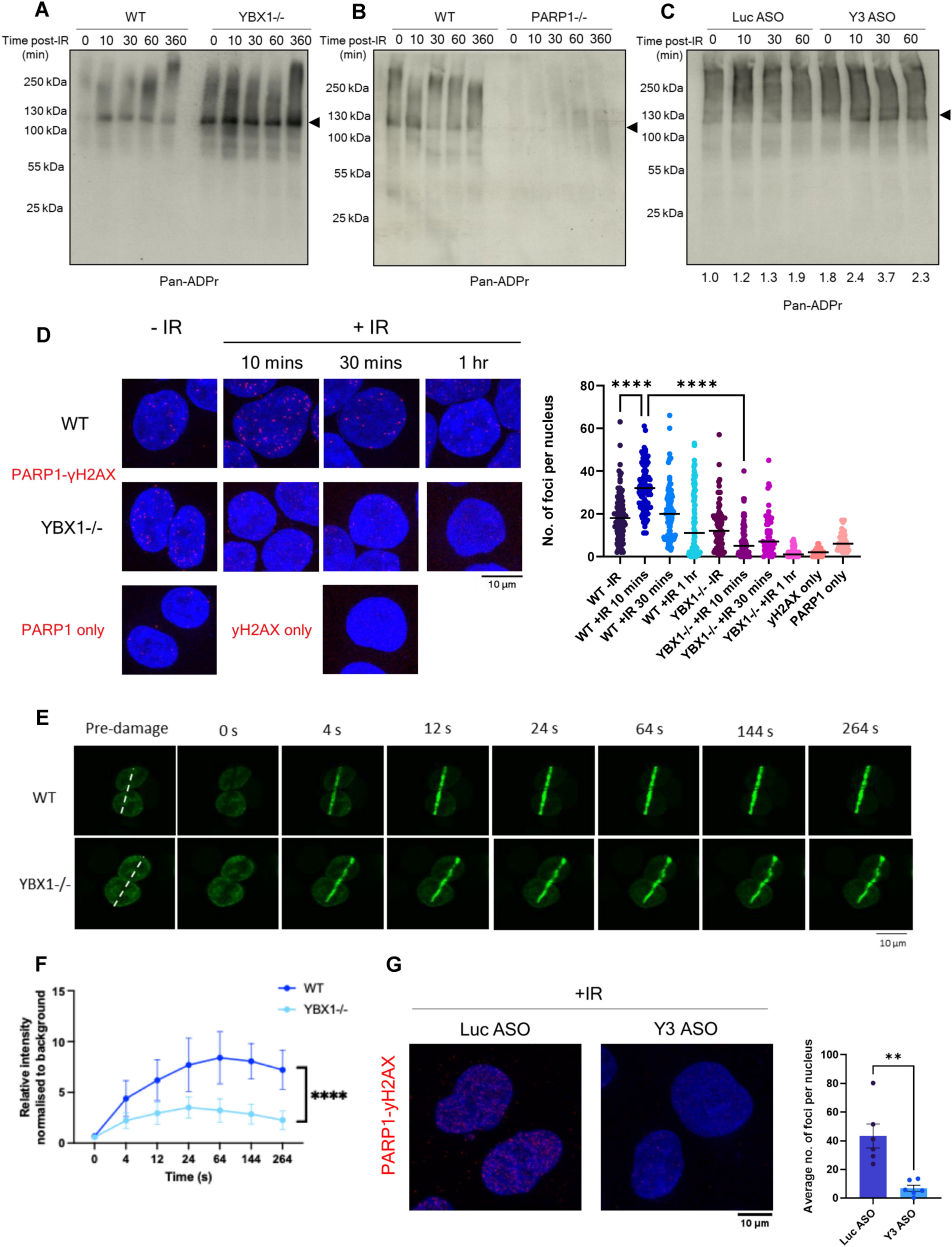
YBX1/ysRNA promote PARP1 residency at DSBs by affecting its auto-modification. (**A**) Western blot for pan-ADP-ribosylation signal in untreated or IR-treated wild-type (WT) or YBX1 knockout (YBX1-/-) HEK293T cells harvested at indicated time points following IR treatment (10 Gy). Bands corresponding to PARP1 auto-modification have been marked with an arrow. (**B**) Western blot for pan-ADP-ribosylation signal in untreated or IR-treated wild-type (WT) or PARP1 knockout (PARP1-/-) HEK293T cells harvested at indicated time points following IR treatment (10 Gy). Bands corresponding to PARP1 auto-modification have been marked with an arrow. (**C**) Western blot for pan-ADP-ribosylation signal in HEK293T cells treated with Y3 or control (Luc) ASO, harvested at indicated time points following IR treatment (10 Gy). Bands corresponding to PARP1 auto-modification have been marked with an arrow. (**D**) PLA of PARP1 and γH2AX in wild type (WT) or YBX1 knockout (YBX1-/-) HEK293T cells, with and without IR treatment (10 Gy, +IR, and -IR, respectively), fixed at indicated time points, including single antibody control assays. Left panel shows representative images, right panel shows quantification carried out in CellProfiler. Statistical significance was determined using Kruskal–Wallis test with Dunn’s multiple comparison, ****P ≤ 0.0001. (**E**) Laser induced DNA damage of wild type (WT) and YBX1-/- HEK 293T cells transiently transfected with YFP-PARP1 plasmid. Representative spinning disc confocal microscopy images before and after the laser induced damage at indicated time points. (**F**) Quantification of relative YFP signal from panel E (N ≥ 10) along the time considered (0–264 s). Mean ± SEM. Statistical significance was determined by two-way ANOVA with multiple comparison test (*P* ≤ 0.0001). (**G**) PLA of PARP1 and γH2AX upon IR treatment (10 Gy, 10 min) of Y3 or control (Luc) ASO-treated HEK293T cells (48 h). Left panel shows representative images, and right panel shows quantification carried out in ImageJ. Mean ± SEM. Statistical significance was determined using Student’s t-test, **P ≤ 0.01.

It was previously suggested that trans-ADP ribosylation of YBX1 dominates over PARP1 auto-modification within YBX1–PARP1 complexes *in vitro* [33, 34]. As PARP1 auto-modification correlates with its dissociation from nucleosome-coupled DNA [54], these findings imply that the proposed stimulation of PARP1 by YBX1 may partially result from a longer retention time of active PARP1 on DNA, due to its reduced auto-modification. We therefore used immunoblotting to investigate levels of PARP1 ADP-ribosylation in cells following IR-induced damage, comparing WT with YBX1-/- cells. Interestingly, we see increased levels of PARP1 auto-ADP-ribosylation when YBX1 is lost (Fig. 6A and Supplementary Fig. S11A), supporting the hypothesis that YBX1 accepting ADP-ribose chains reduces PARP1 auto-modification. As a control, we performed the same experiment in cells lacking PARP1 (Fig. 6B and Supplementary Fig. S11B and C) to confirm that the signal indeed corresponds to PARP1 auto-modification. Similar results were also obtained when ADP-ribosylation levels were investigated in cells depleted of Y3 (Fig. 6C and Supplementary Fig. S11D), supporting co-operation between YBX1 and ysRNA in regulating PARP1 activity. In order to establish whether this alteration in PARP1 auto-modification held relevance to its residency at DSBs, we performed PLA using antibodies against PARP1 and γH2AX as a measure of PARP1 localisation at DSBs. At 10 minutes post-IR treatment, we see a significantly higher number of PLA foci compared with untreated control in WT cells, but this is not seen in YBX1-/- cells (Fig. 6D). This suggests the presence of YBX1 allows for PARP1 to be maintained in proximity to γH2AX and, by extension, DSBs for longer. To support this observation, we then performed laser microirradiation in HEK293T cells transiently expressing YFP-tagged PARP1, in order to visualise its recruitment to DSBs in both WT and YBX1-/- cells. Interestingly, upon YBX1-/- we observe significantly reduced PARP1 signal at sites of laser-induced DSBs (Fig. 6E and F). This suggests that in the absence of YBX1, PARP1 dissociates from damaged chromatin more rapidly, thus supporting our suggestion that YBX1 can influence PARP1 residency at damage sites. Similarly, co-localisation of PARP1 and γH2AX is also reduced in cells treated with Y3 ASO, as observed by a significantly lower number of PLA foci with Y3 ASO treatment compared with control (Fig. 6G). Together, these data suggest that YBX1 and Y3 5′ ysRNA work in combination to promote PARP1 residency at DSBs via reduced auto-modification.

## Discussion

Radioprotective bystander effects transmitted by exosomes have been previously studied, largely in terms of their influence on cell survival [10, 11]. While one study did suggest RNA involvement and enhanced DNA repair as a potential mechanism, they did not identify any specific candidate RNA species which may be contributing to this phenotype [10]. Here, we show that radioprotection is YBX1-dependent and is driven, at least partially, by Y3-derived ysRNA. It is possible that other species of sncRNA which were identified in our sequencing dataset but not explored (e.g. miRNAs or tRFs) may further enhance radioprotection. However, we show that similar reductions in DNA damage markers could be induced by both ysRNA and DD EVs, alongside an influence on overall cell survival in response to IR treatment, supporting the significant involvement of ysRNA in radioprotection. While there was also a visible reduction in cell survival observed upon Y3 depletion in untreated cells, this impact is augmented upon IR treatment. The contribution of Y RNA to survival in untreated conditions is likely due to the reported functionality of Y3 RNA in cell proliferation, underpinned by its modulation of initiation at DNA replication forks [55, 56]. Despite the currently unknown functions of ysRNA species, it is possible that they may act in similar ways to the full-length Y RNAs from which they derive.

In line with this, we show that Y3-derived ysRNA becomes localised to the nucleus specifically upon IR-induced DNA damage, consistent with previous reports of full-length Y3 RNA accumulating in nuclei and contributing to survival following UV-induced damage [26]. While it was suggested that dissociation from Y RNA permitted nuclear import of Ro60 [25], the mechanisms underlying the translocation of Y RNA itself are yet to be fully determined. Nuclear-cytoplasmic shuttling of the Ro60/Y3 RNA complex has, however, been linked to the Zipcode-Binding Protein, ZBP1. Interestingly, both Ro60 and ZBP1 could also be co-purified with YBX1 [57], supporting our suggestion of proximity between YBX1 and Y3 RNA within the nucleus. In addition, we show that YBX1 contributes to the stability of nuclear Y3 5′ ysRNA, further supporting their interaction. Due to the activity of YBX1 as an m5C reader [51], we also investigated whether this species could be methylated, given that m5C recognition has been suggested to be important for DNA repair-related functions of YBX1 [58]. Interestingly, full length Y1 RNA has been detected among NSUN2 targets in multiple studies [59, 60], supporting our observation of NSUN2-mediated methylation of Y3 5′ ysRNA, given the sequence similarity between Y1 and Y3 RNAs. Nevertheless, we did not observe a significant interaction between Y1 5′ ysRNA and YBX1 in our study. Therefore, further investigation would be required to delineate the importance of ysRNA methylation for its DDR role.

Our study demonstrates that ysRNA involvement in radioprotection and, by extension, canonical DNA repair, is dependent on YBX1. While YBX1 is widely regarded as a DNA repair factor, the full extent of its involvement in the repair process is still under study and likely encompasses multiple mechanisms [61]. Many reports suggest the DNA repair functionality of YBX1 can be underpinned by RNA binding, in particular that of long non-coding RNAs [62–64]. For example, YBX1 binding to Aerrie was shown to impact DNA damage signalling in endothelial cells [62]. Additionally, interaction between DARS-AS1 and YBX1 was suggested to increase radioresistance of glioblastoma cells via post-transcriptional regulation of target genes, including homologous recombination factors [63]. On the other hand, YBX1 has also been shown to directly interact with known DSB repair proteins, including the MRN complex, Ku80 and WRN [19, 65], supporting its localisation at DSBs. Of note, YBX1 was also identified to interact with PARP1 *in vitro* and stimulate its activity by acting as a preferential PAR acceptor within YBX1–PARP1–DNA ternary complexes [33, 34]. As PARP1 is a key contributor to DSB repair, we focused on the possible role of YBX1 as a PARP1 stimulating factor. PARP1 and deposited ADP-ribose chains are known to play important roles in DNA repair, including contributions to chromatin reorganisation and downstream repair factor recruitment [35]. While our data show colocalisation between YBX1, ysRNA and PARP1 at DSB sites, it has been suggested that YBX1 can participate in phase transitions initiated by poly(ADP-ribose) chains, potentially enabling its DNA repair function [66]. This is supported by other studies proposing phase separation to occur at DSB sites and contribute to efficient repair [67–69]. However, YBX1 also contains an intrinsically disordered domain at its C-terminus and has been reported to undergo liquid-liquid phase separation [17]. Therefore, it is possible that the association we observe between Y3-derived ysRNA, YBX1 and PARP1 may result from a similar phase separation event, rather than a direct interaction and complex formation.

Nevertheless, we demonstrate, for the first time, PARP1-dependent ADP-ribosylation of ysRNA. While ADP-ribosylation of RNA has been previously reported, this was attributed to other members of the PARP family, such as PARP10, PARP11 and PARP15 [70]. When tested in cells, RNA ADP-ribosylation was found to be responsive to various stressors, including H_2_O_2_ (a known activator of PARP1 activity), although this did not appear to impact modification of small RNA populations [71]. Other proposed roles of RNA ADP-ribosylation include initiation of immune responses and influencing mRNA stability [39, 70]. While PARP1 has not been directly implicated in RNA ADP-ribosylation, it has been shown to modify phosphorylated DNA ends, which was suggested to potentially promote DNA repair [39]. Furthermore, PARP1 is able to bind various types of RNA, including mRNA, miRNA, small nucleolar RNA and lncRNA [40, 41], with a suggested preference for binding GC-rich sequences [40]. Although further investigation is required to determine the exact functions and importance of nucleic acid ADP-ribosylation, we suggest that the observed ysRNA ADP-ribosylation within PARP1–YBX1 complexes, and the resulting decrease in PARP1 auto-modification, may result in increased chromatin residency of PARP1. As a result, active PARP1 remains present at DNA lesions for longer, promoting increased deposition of ADP-ribose chains to facilitate repair. This suggests a previously unknown contribution of ysRNA to DSB repair.

**Figure 7.**
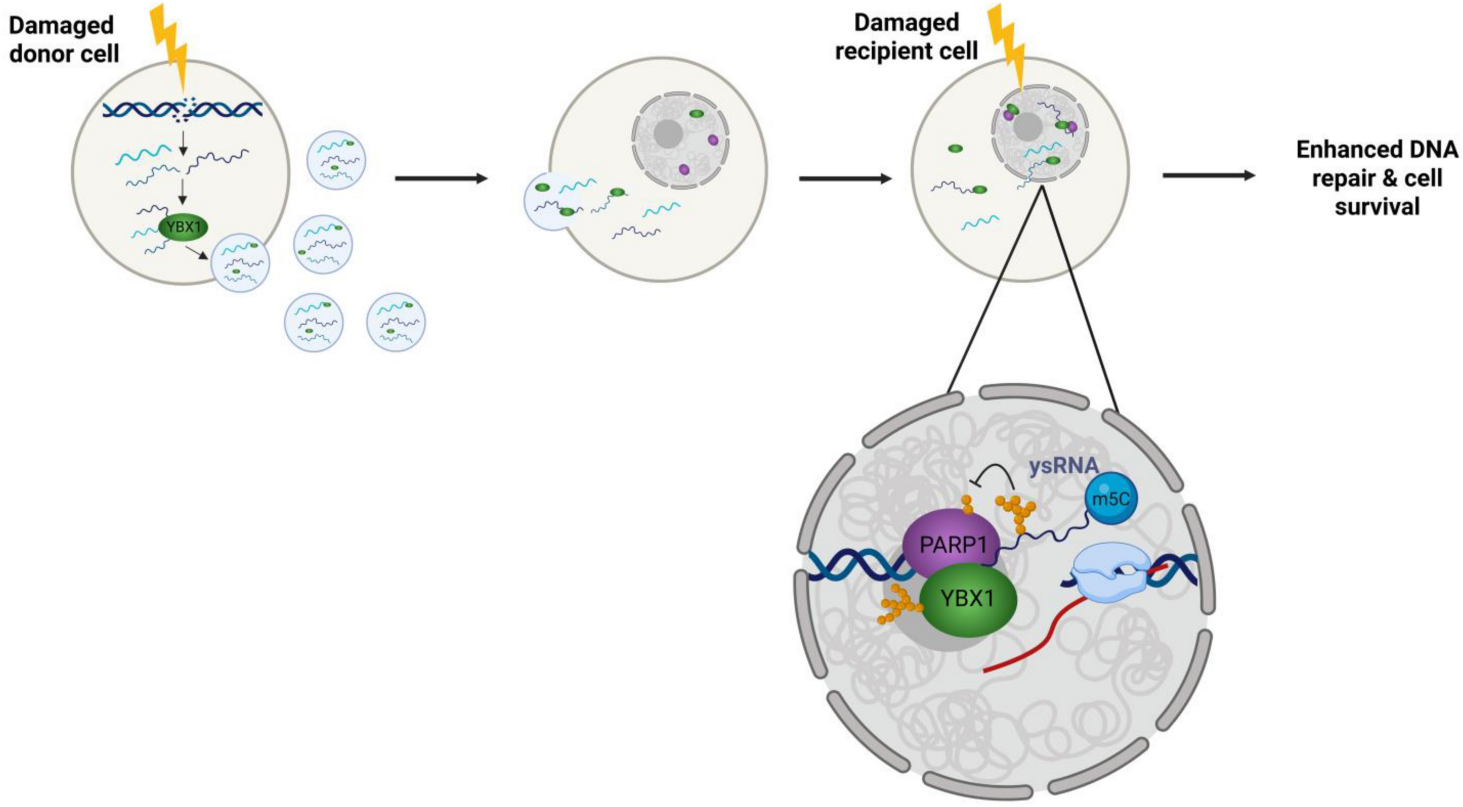
Proposed model of YBX1-ysRNA mediated radioprotective bystander phenotype. YsRNA fragments are loaded into exosomes by YBX1 and can be taken up by recipient cells, where they modulate PARP1 residency on chromatin by serving as its substrate and preventing PARP1 auto-modification. Generated in Biorender.

Overall, we propose a model whereby YBX1 plays a dual role in mediating radioprotective bystander responses via Y3-derived ysRNA. First, YBX1 packages ysRNA into exosomes which can be transmitted to bystander cells and enhance DNA repair and cell survival in response to subsequent radiation insult. Secondly, YBX1 binds methylated ysRNA in the nuclei of these cells, facilitating complex formation with PARP1. This allows for ADP-ribosylation of ysRNA and YBX1, reducing PARP1 auto-modification and leading to increased PARP1 residency on chromatin to facilitate efficient DNA repair (Fig. 7). These results provide important insights for a greater understanding of RNA-dependent DDR and provide a new perspective on regulation of PARP1 activity, which is highly relevant to cancer therapy.

## Supplementary Material

gkaf517_Supplemental_File

## Data Availability

Exosome Small RNA-seq data have been deposited to GEO under GSE269346. Any additional information required to re-analyse the data reported in this work paper is available from the lead contact upon request.
