# Supplementary material for: Non-coding Y RNA fragments in a complex with YBX1 modulate PARP1 residency at DNA double strand breaks
Source: Nucleic Acids Res. 2025 Jun 18;53(11):gkaf517. doi: 10.1093/nar/gkaf517 (PMC12203914; doi:10.1093/nar/gkaf517)
Supplement: gkaf517_Supplemental_File [file gkaf517_supplemental_file.pdf]

Supplementary Figure 1

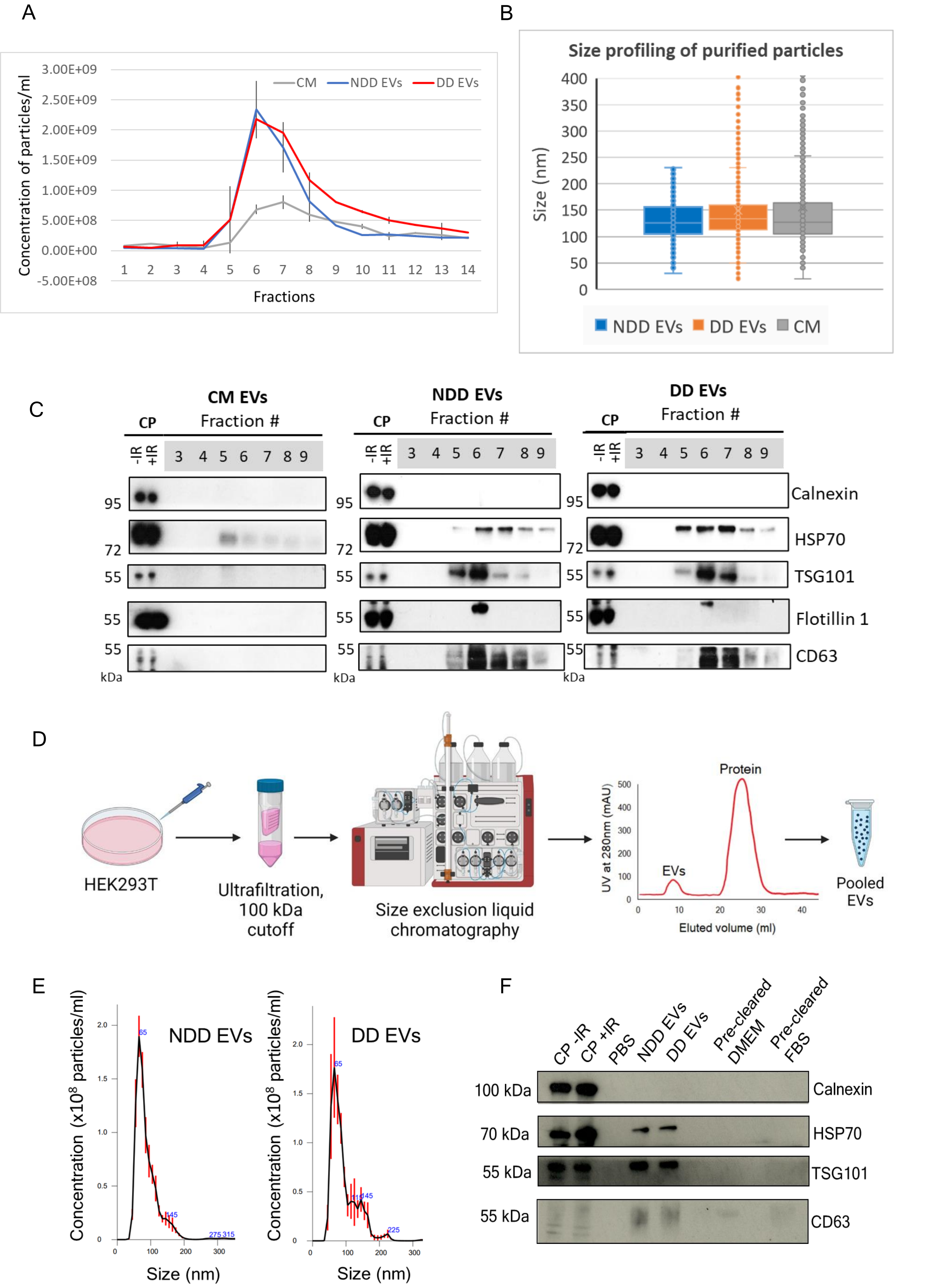

Supplementary Figure 2

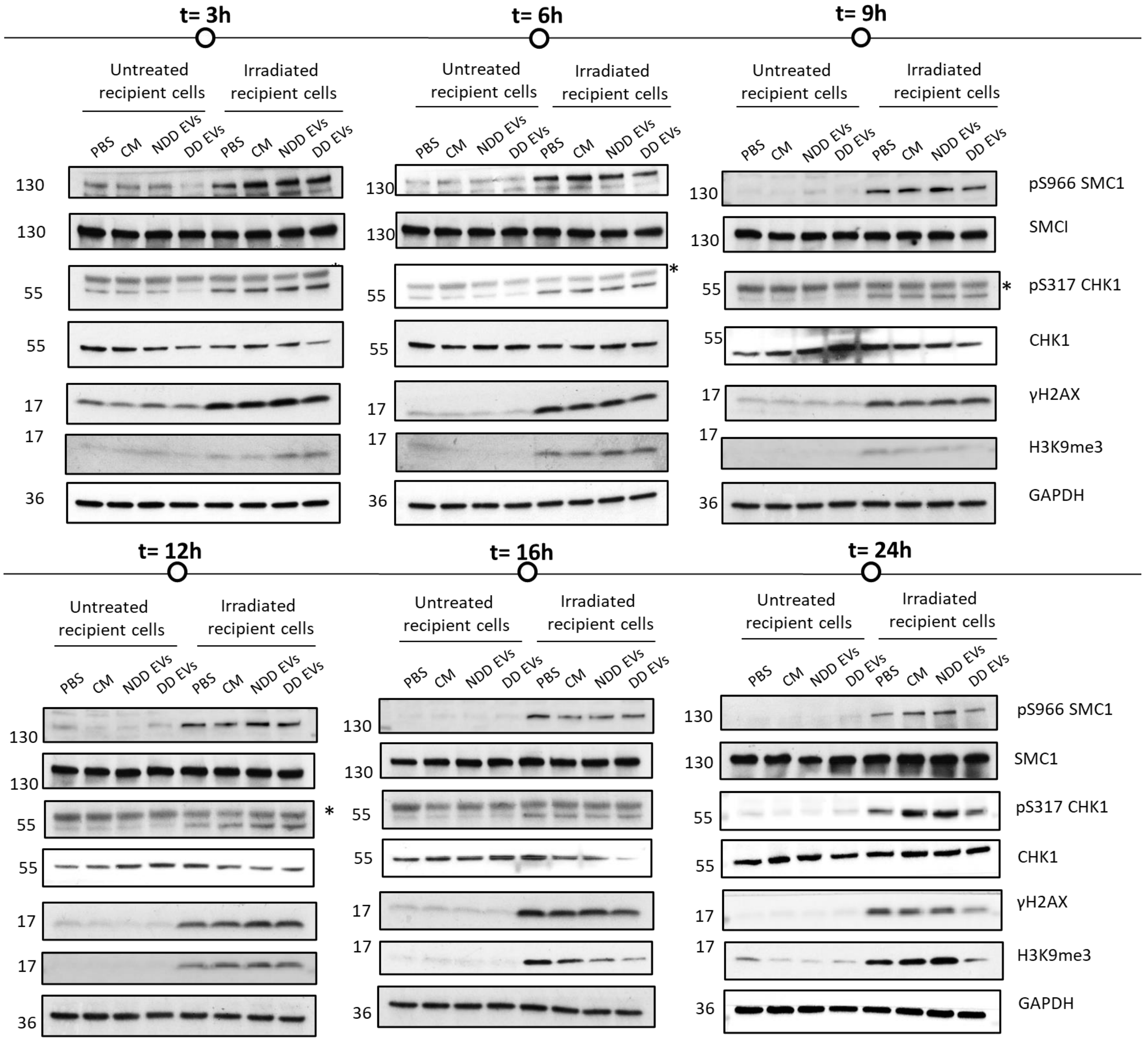

Supplementary Figure 3

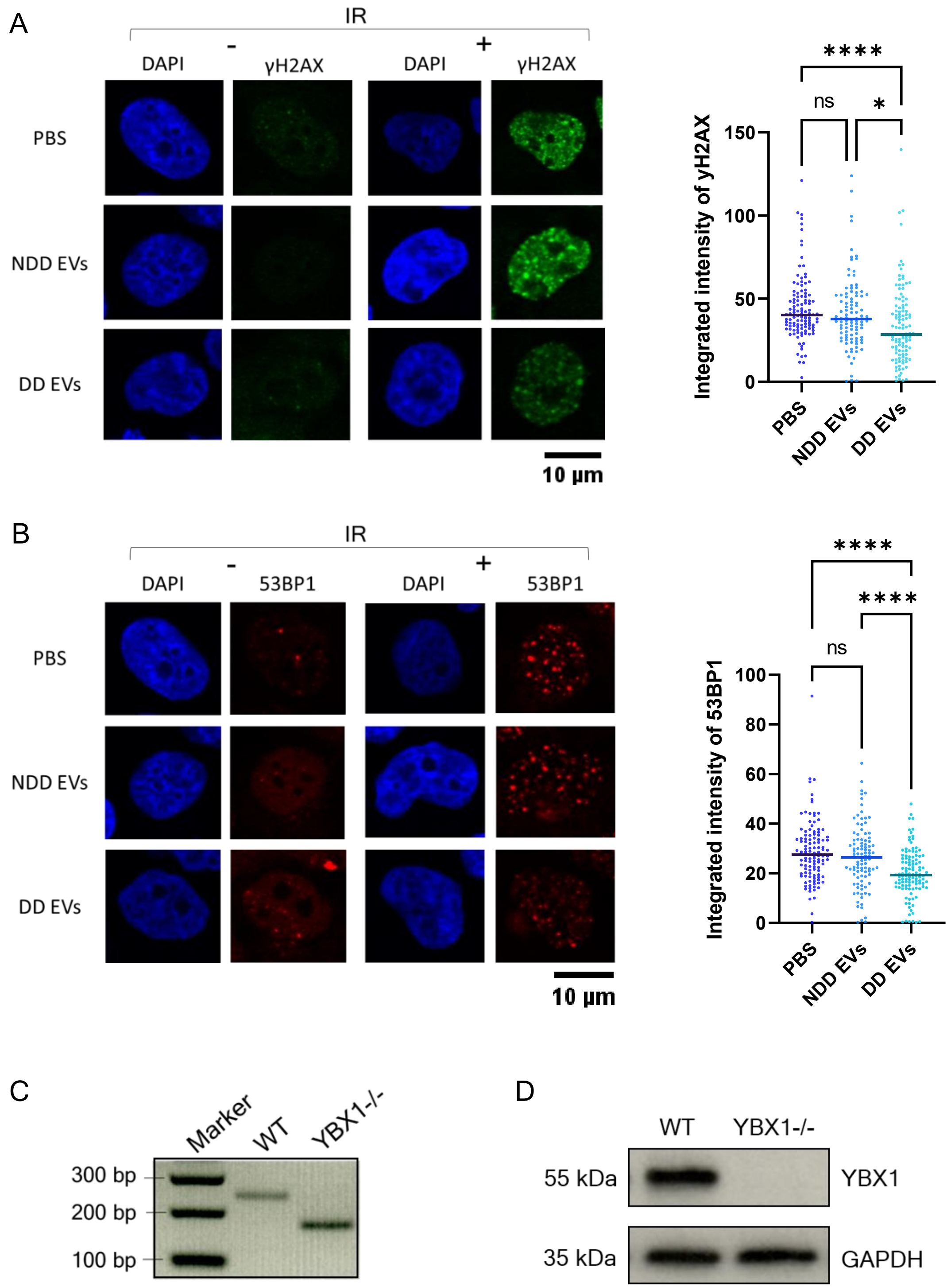

Supplementary Figure 4

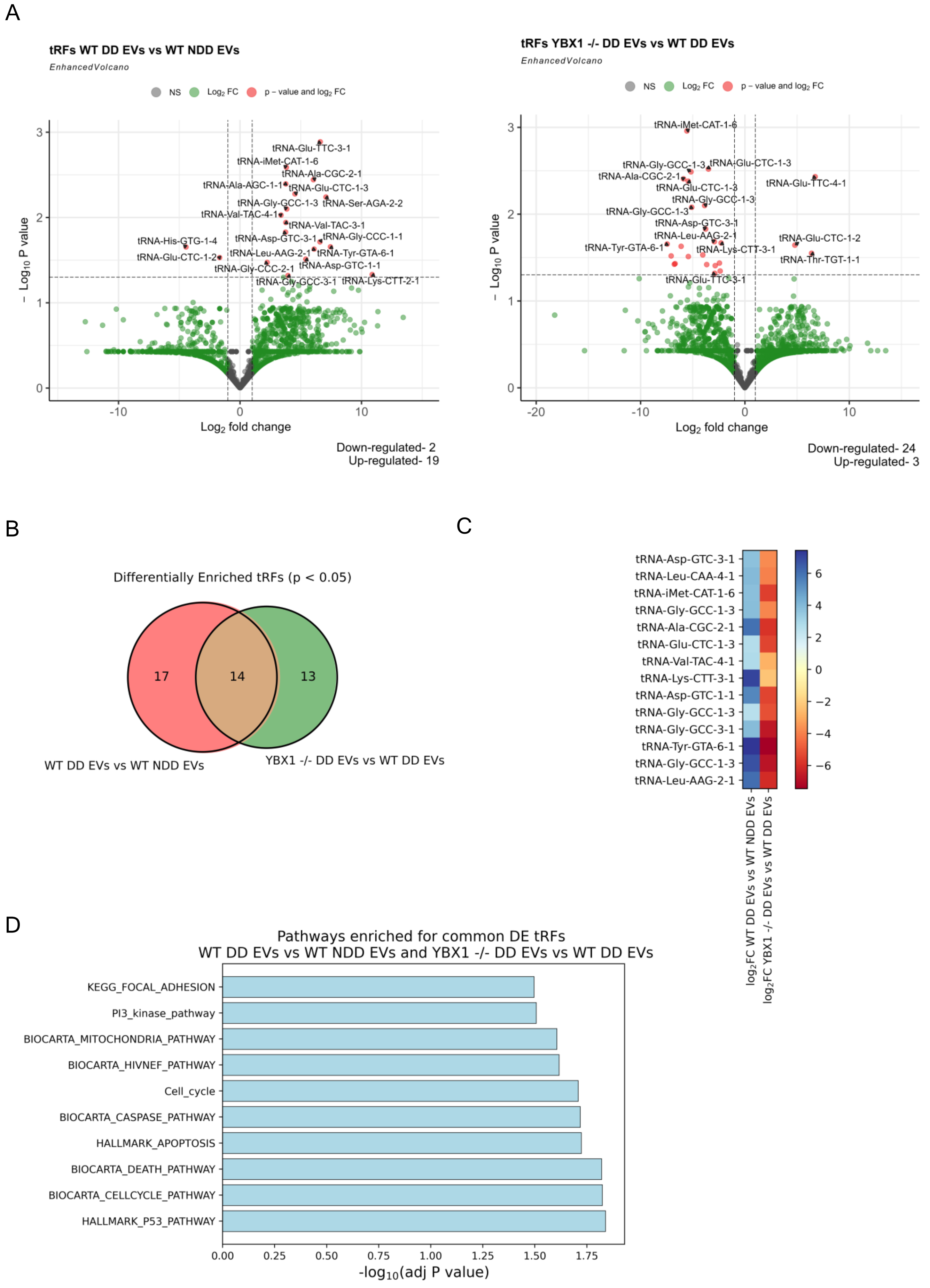

Supplementary Figure 5

A

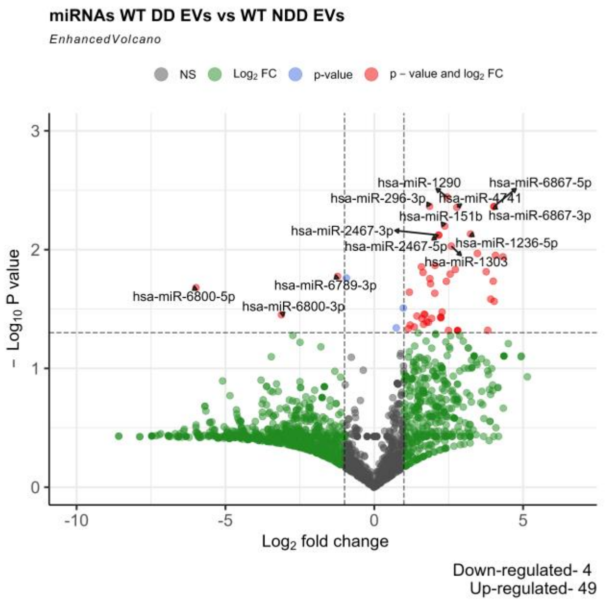

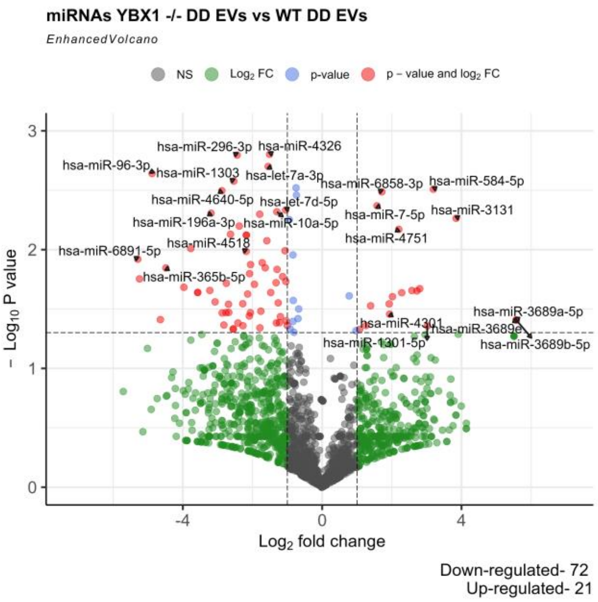

B

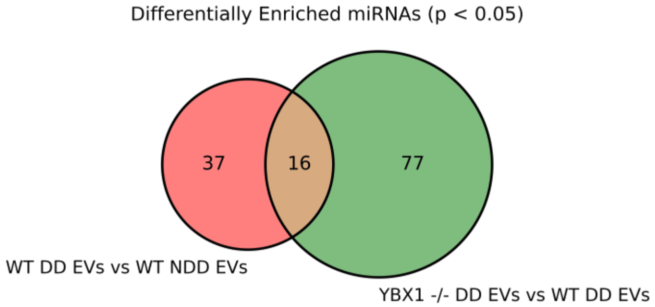

C

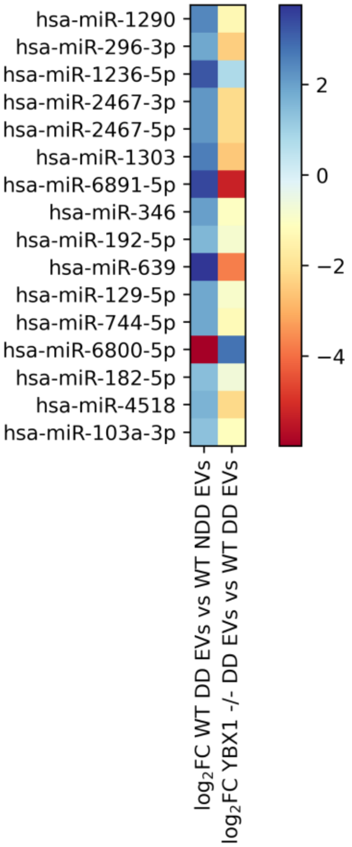

D

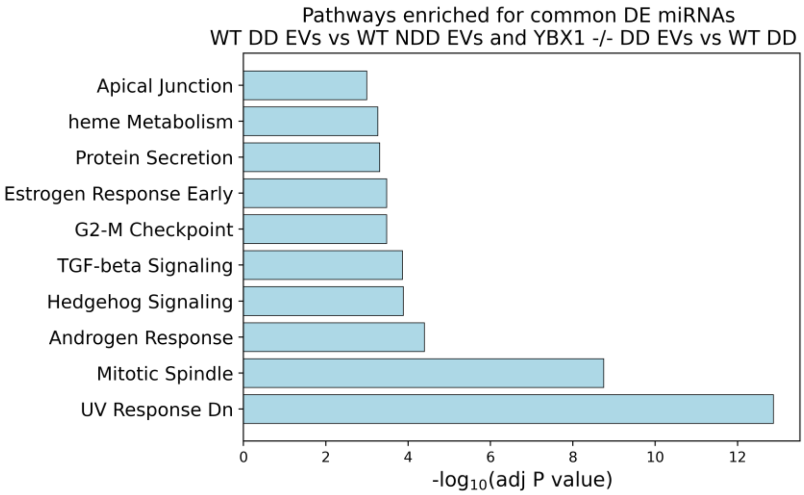

Supplementary Figure 6

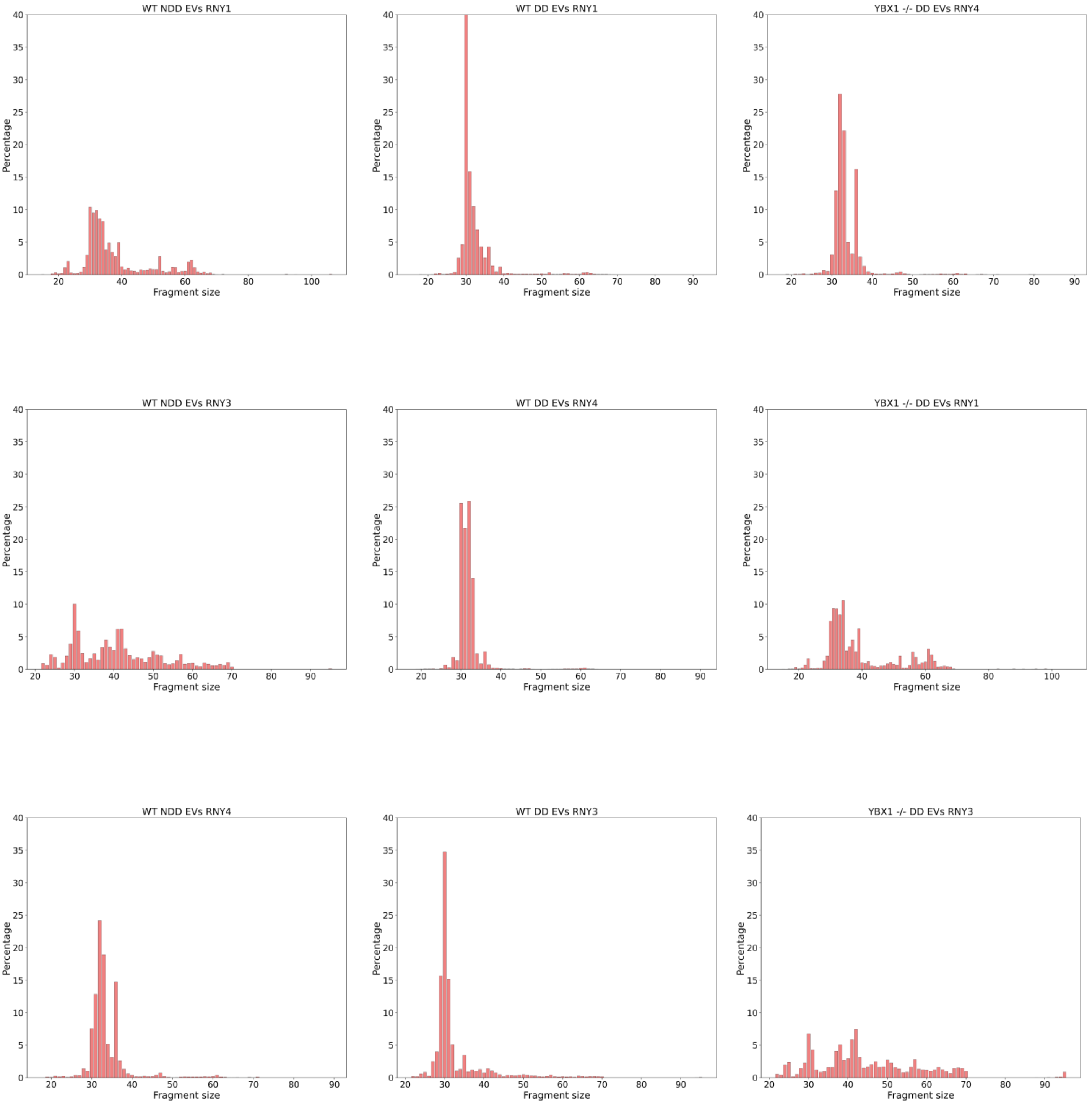

Supplementary Figure 7

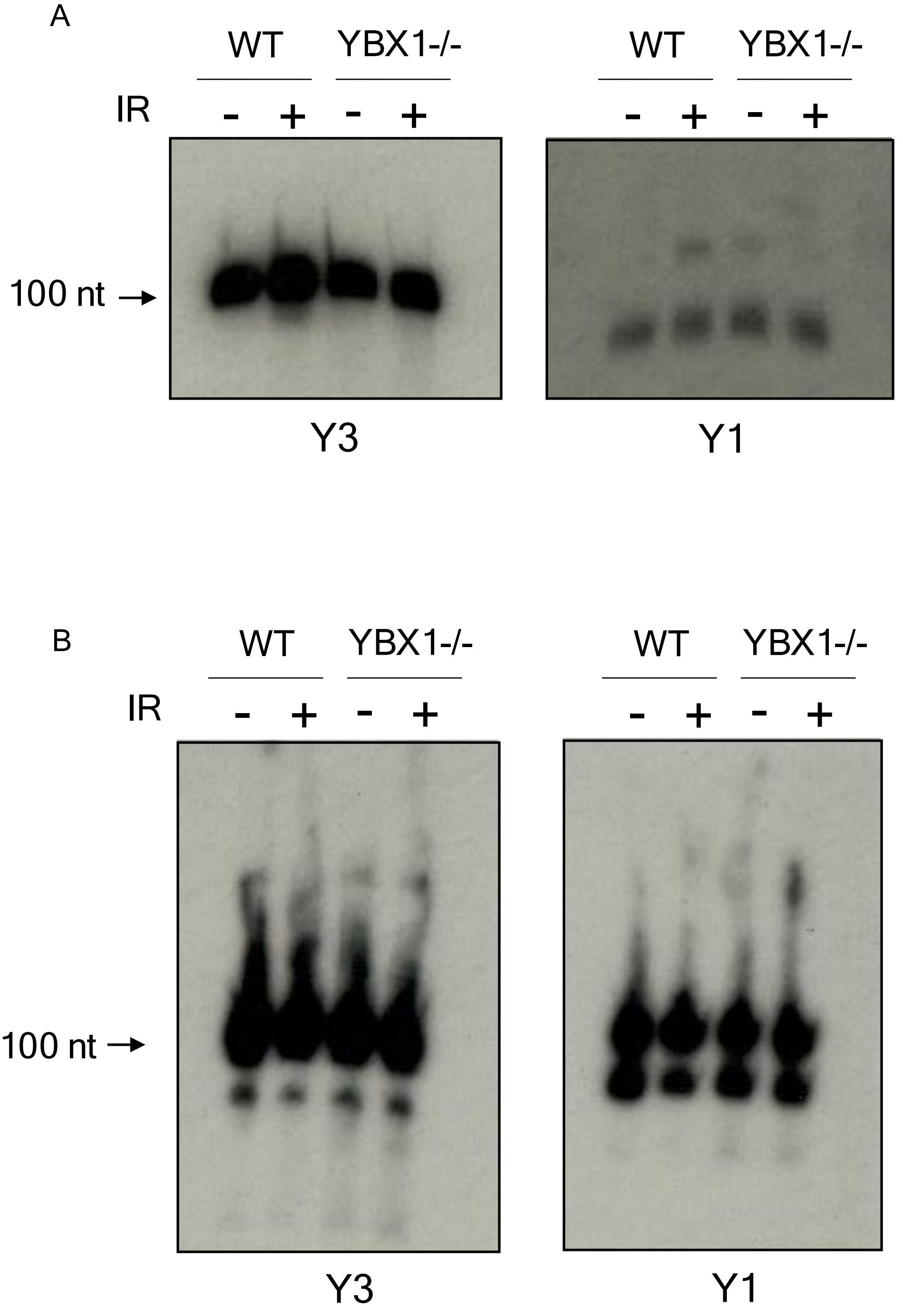

Supplementary Figure 8

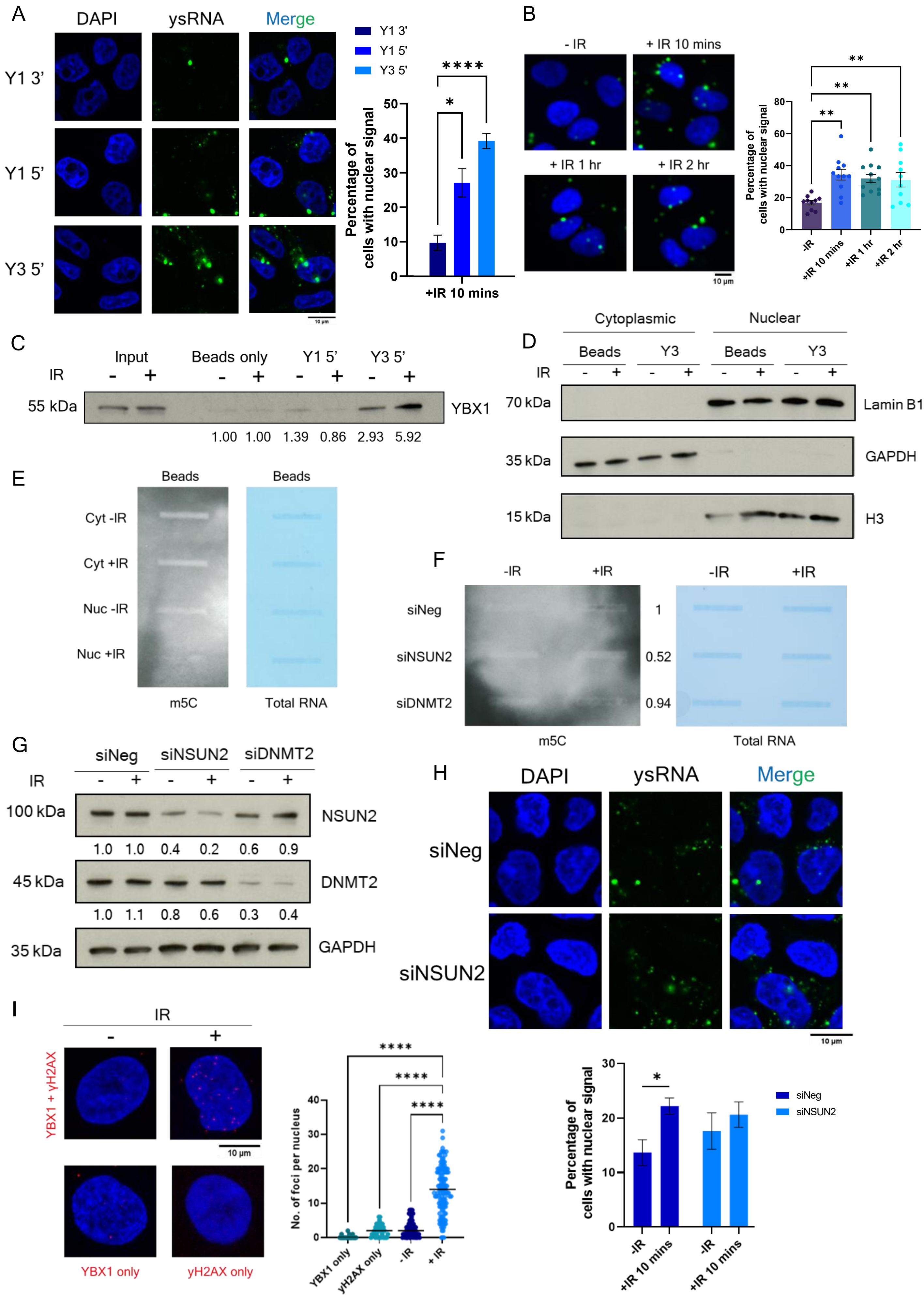

Supplementary Figure 9

A

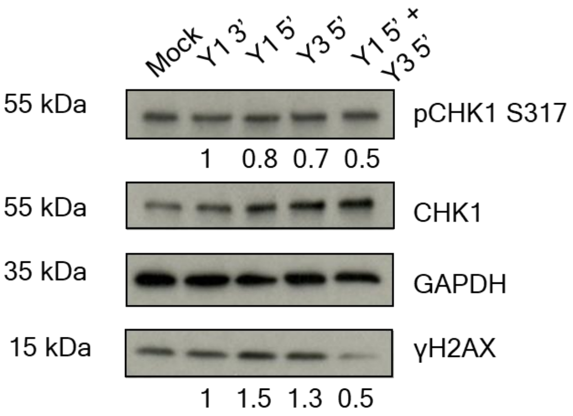

B

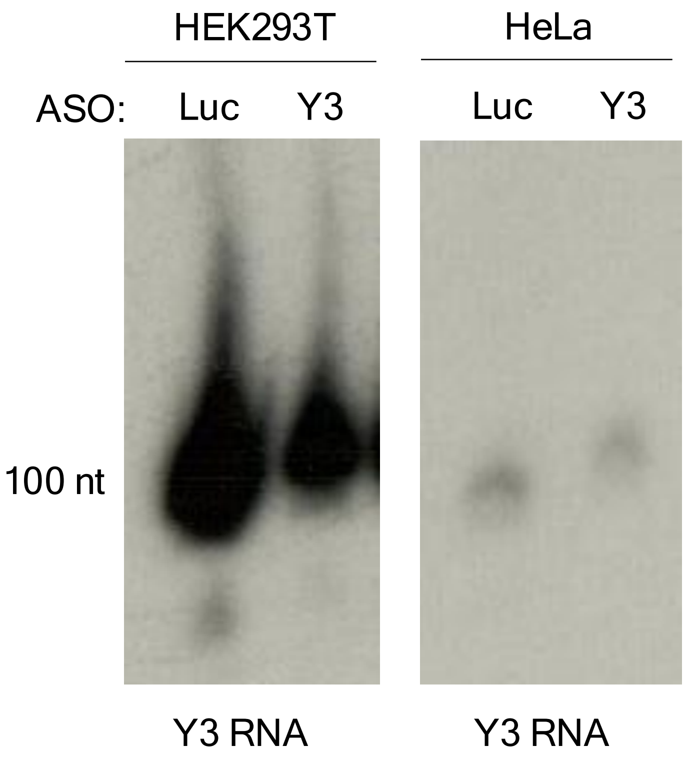

Supplementary Figure 10

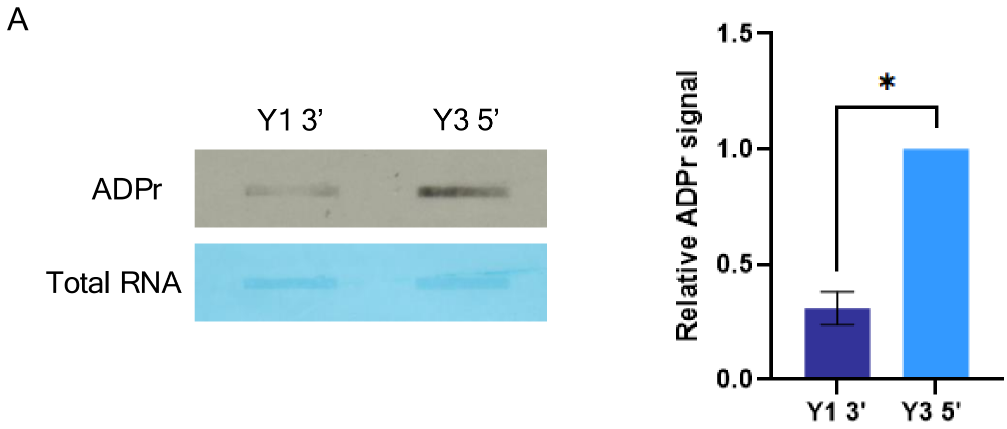

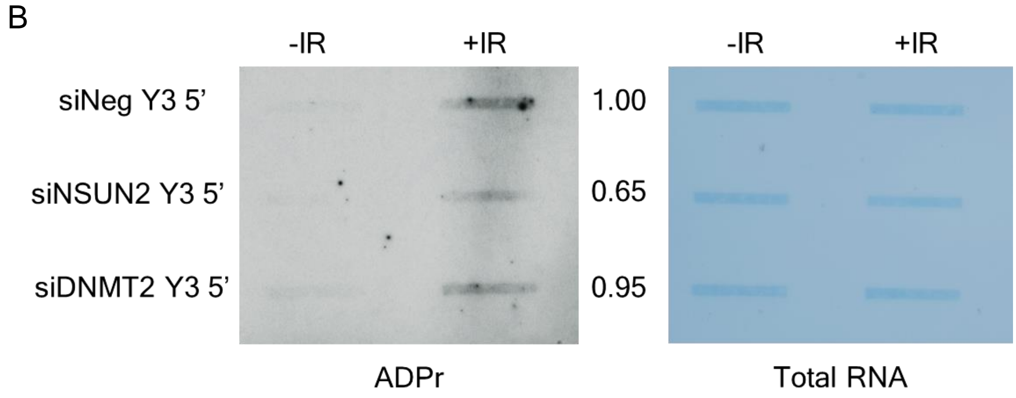

Supplementary Figure 11

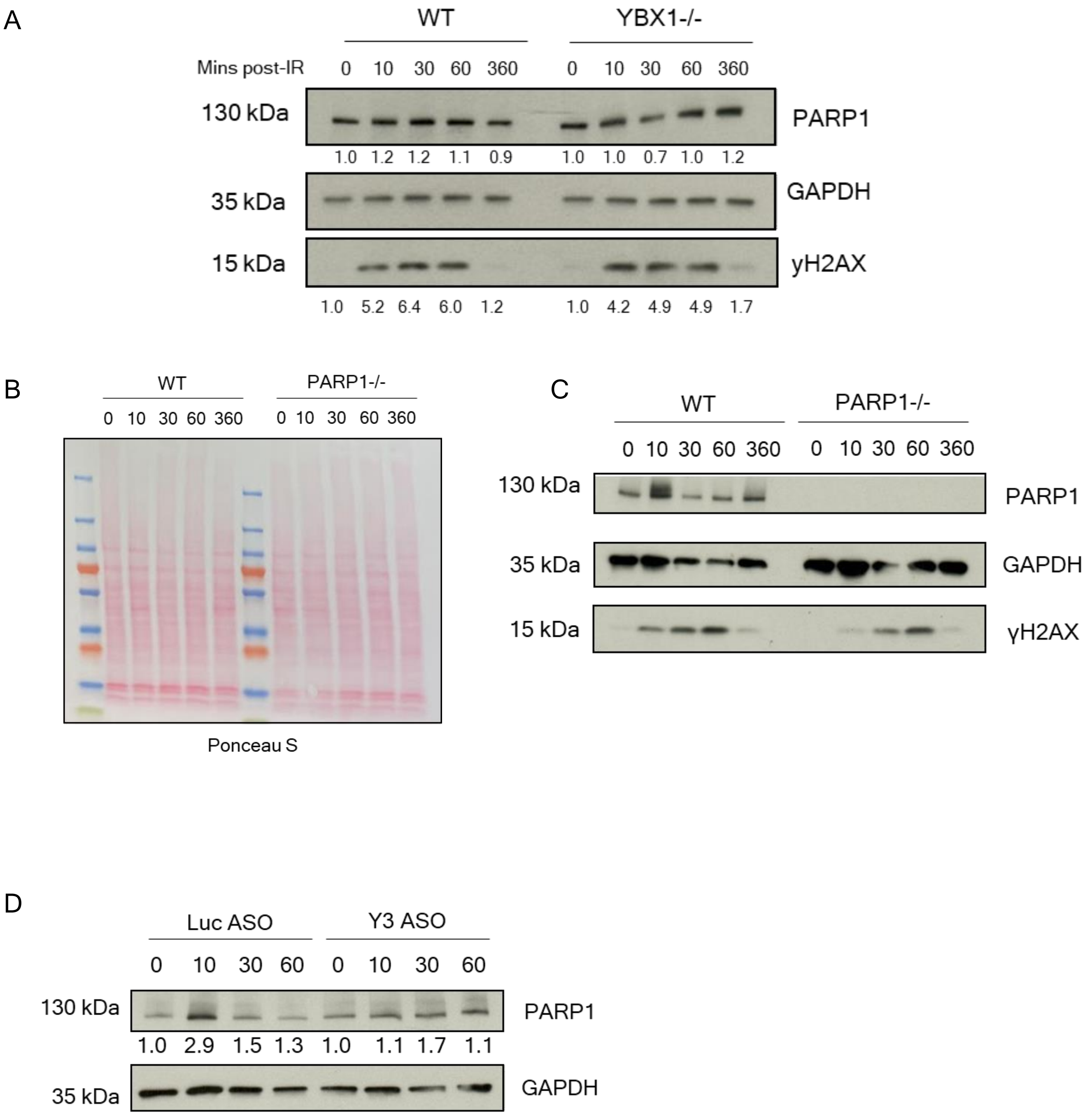

Supplementary Figure 1

A) Concentration of particles present in fractions (1-14) obtained by size exclusion chromatography of clear medium (CM) or cell culture media from untreated (NDD EVs) or 10 Gy irradiated (DD EVs) HEK293T cells, measured by Nanoparticle Tracking Analysis.

B) Size distribution of particles present in fractions 3-9 obtained by size exclusion chromatography of clear medium (CM) or cell culture media from untreated (NDD EVs) or 10 Gy irradiated (DD EVs) HEK293T cells, measured by Nanoparticle Tracking Analysis.

C) Quality control Western blot showing exosome-specific markers, HSP70, TSG101, Flotillin 1 and CD63, and cellular marker, Calnexin, in fractions obtained by size exclusion chromatography of clear medium (CM) or cell culture media from untreated (NDD EVs) or 10 Gy irradiated (DD EVs) HEK293T cells, and cellular protein (CP) control.

D) Workflow for automated size exclusion chromatography, generated in Biorender. Briefly, cell culture media was isolated, concentrated by ultrafiltration and loaded onto a size exclusion liquid chromatography column, connected to a UV flow cell. EV-containing fractions were collected and pooled on the basis of UV absorbance (fractions 5-9).

E) Concentration and size distribution of particles in pooled EV samples from non-damage-derived (NDD EVs) and damage-derived exosomes (DD EVs) obtained by automated size exclusion chromatography.

F) Quality control Western blot showing exosome-specific markers, HSP70, TSG101 and CD63, and cellular marker, Calnexin, in pooled EV samples obtained by size exclusion chromatography, alongside cellular protein (CP), pre-cleared DMEM and pre-cleared FBS controls.

Supplementary Figure 2

Western blots showing optimisation of time (t) for DNA damage response-related effects to be visible following following transfer of exosomes isolated from a clear media only control (CM), untreated HEK293T cells (NDD EVs), irradiated HEK293T cells (DD EVs) or a PBS only negative control, to recipient cells. * indicates non-specific bands. Panel of DNA damage associated markers shown, chosen based on varying contributions to the DNA damage response.

Supplementary Figure 3

A) Representative immunofluorescence images (left) and quantification (right) showing levels of γH2AX following transfer of non-damage-derived exosomes (NDD EVs), damage-derived exosomes (DD EVs) or a PBS control in IR-treated (10 Gy, 6 h) and untreated HEK293T cells. Nuclear signal intensity was quantified using CellProfiler, $N \geq 100$ cells. Statistical significance determined by Kruskall-Wallis test with Dunn's multiple comparison. $*p \leq 0.05$, $****p \leq 0.0001$.

B) Representative immunofluorescence images (left) and quantification (right) showing levels of 53BP1 foci following transfer of NDD EVs, DD EVs or a PBS control in IR-treated (10 Gy, 6 h) and untreated HEK293T cells. Nuclear signal intensity was quantified using CellProfiler, $N \geq 100$ cells. Statistical significance determined by Kruskall-Wallis test with Dunn's multiple comparison, $****p \leq 0.0001$.

C) Analysis of wild-type (WT) and YBX1 -/- HEK293T cells by PCR flanking the genomic target site of CRISPR-Cas9-mediated editing to confirm knockout.

D) Western blot confirming YBX1 knockout, with GAPDH as a loading control.

Supplementary Figure 4

A) Volcano plots showing differentially expressed tRNA-derived fragments (tRFs) in exosomes upon damage (left) and YBX1 knockout (right). The red points show up- and down-regulated tRNA-corresponding reads with $\log2FC > 1$ and $\log2FC < -1$ and $P\text{-adj} < 0.001$, respectively.

B) Venn diagram showing the number of commonly differentially expressed tRFs between damage and YBX1 knockout conditions.

C) Heat map of common differentially expressed tRFs identified in b), showing their log2FC in damage vs non-damage-derived exosomes, and YBX1 knockout vs wild-type damage-derived exosomes, respectively.

D) Pathways identified by Geneset enrichment analysis as affected by the differentially expressed tRFs whose presence in exosomes is damage and YBX1-dependent.

Supplementary Figure 5

A) Volcano plots showing differentially expressed miRNA species in exosomes upon damage (left) and YBX1 knockout (right). The red points show up- and down-regulated miRNA-corresponding reads with $\log2FC > 1$ and $\log2FC < -1$ and $P\text{-adj} < 0.001$, respectively.

B) Venn diagram showing the number of commonly differentially expressed miRNA species between damage and YBX1 knockout conditions.

C) Heat map of common differentially expressed miRNA species identified in b), showing their log2FC in damage vs non-damage-derived exosomes, and YBX1 knockout vs wild-type damage-derived exosomes, respectively.

D) Pathways identified by Geneset enrichment analysis as affected by the differentially expressed miRNA species whose presence in exosomes is damage and YBX1-dependent.

Supplementary Figure 6

Size distribution of reads corresponding to Y RNA genes (Y1, Y3 and Y4) from WT NDD EVs, WT DD EVs and YBX1-/- DD EVs from left to right, respectively.

Supplementary Figure 7

A) Northern blot showing levels of Y3 and Y1 RNA in wild-type (WT) and YBX1 knockout (YBX1-/-) cells which were untreated (-) or subjected to 10 Gy IR for 48 h (+).

B) Northern blot showing levels of Y3 and Y1 RNA and their associated degradation products in wild-type (WT) and YBX1 knockout (YBX1-/-) cells which were untreated (-) or subjected to 10 Gy IR for 48 h (+).

Supplementary Figure 8

A) Representative images (left) and quantification (right) of nuclear localisation of AlexaFluor488-labelled ysRNA oligonucleotides following transfection into HEK293T cells (24 h) and ionising radiation (IR)-treatment (10 Gy, 10 mins). Quantification was carried out using ImageJ and represented as the percentage of cells per frame with nuclear fluorescent signal ($N \geq 6$ frames, at least 70 cells per condition). Statistical significance was determined using two-way ANOVA with Bonferroni's multiple comparison, *$p \leq 0.05$, ****$p \leq 0.0001$.

B) Representative images (left) and quantification (right) of nuclear localisation of AlexaFluor488-labelled Y3 5' ysRNA oligonucleotides following transfection into U2OS cells (24 h) and ionising radiation (IR)-treatment (10 Gy, indicated times). Quantification was carried out using ImageJ and

represented as the percentage of cells per frame with nuclear fluorescent signal ($N \geq 10$ frames, at least 100 cells per condition). Mean ± SEM. Statistical significance was determined using one-way ANOVA with Dunnett's multiple comparison, **$p \leq 0.01$.

C) Co-immunoprecipitation of YBX1 with biotinylated Y3 5' or Y1 5' ysRNA oligonucleotide, or beads only control (no RNA) from HEK293T whole cell lysates. Numbers represent fold change in band intensity compared with beads only control, normalised to input. Quantified using ImageJ.

D) Western blot for cytoplasmic marker, GAPDH, and nuclear markers, Lamin B1 and histone H3 in samples used for RNA immunoprecipitation and slot blot.

E) Slot blot for m5C modification (left) and total RNA stain (right) in beads only control samples following RNA immunoprecipitation from irradiated (10 Gy, +IR) or unirradiated (-IR) cytoplasmic (Cyt) or nuclear (Nuc) cell fractions.

F) Slot blot for m5C modification (left) and total RNA stain (right) following pulldown of biotinylated Y3 5' ysRNA oligonucleotide after incubation with nuclear fractions from IR-treated (10 Gy, 10 mins, +IR) and untreated (-IR) cells, which had been subjected to siRNA-mediated knockdown of NSUN2 or DNMT2 (60 nM, 48 h) or treated with a negative control siRNA (siNeg). Numbers represent signal intensity of m5C in Y3 5' pulldown, normalised to corresponding total RNA signal. Quantified using ImageJ.

G) Western blot for NSUN2 and DNMT2 expression, with GAPDH as a loading control, in samples used for RNA immunoprecipitation and slot blot. Numbers represent fold change in normalised band intensity over control, quantified using ImageJ.

H) Representative images (top) and quantification (bottom) of nuclear localisation of AlexaFluor488-labelled Y3 5' oligonucleotides following transfection into HEK293T cells treated with negative control siRNA (siNeg) or siRNA against NSUN2 (siNSUN2) (60 nM, 48 h) and ionising radiation (IR)-treatment (10 Gy, 10 mins). Mean ± SEM. Quantification was carried out using ImageJ and represented as the percentage of cells per frame with nuclear fluorescent signal ($N \geq 6$ frames, at least 80 cells analysed). Statistical significance was determined using two-way ANOVA with Bonferroni's multiple comparison, *$p \leq 0.05$.

I) PLA of YBX1 and γH2AX, with and without IR treatment (10 Gy, +IR and -IR, respectively), including single antibody control experiments. Left panel shows representative images, right panel shows quantification carried out in CellProfiler. $N \geq 100$ cells. Statistical significance was determined using Kruskall-Wallis test with Dunn's multiple comparison. ****$p \leq 0.0001$.

Supplementary Figure 9

A) Western blot showing expression levels of pCHK1 S317 and γH2AX, with CHK1 and GAPDH as loading controls, following transfection with various synthetic ysRNA oligonucleotides (Y1 3', Y1 5', Y3 5' or a combination of Y1 5' and Y3 5', 10 nM for 24 h) or mock transfected control upon IR treatment (10 Gy, 2 h). Numbers represent fold change in normalised band intensity over control, quantified using ImageJ.

B) Northern blot showing Y3 RNA levels upon control (Luc) and Y3 ASO treatment (125 nM, 48 h) in HEK293T and HeLa cells.

Supplementary Figure 10

A) Slot blot for ADPr modification (left) and associated quantification (right) following pulldown of biotinylated control Y1 3' and Y3 5' oligonucleotides, after incubation with nuclear fractions from IR-treated (10 Gy, 10 mins). ADPr signal intensity was quantified using ImageJ and normalised to corresponding total RNA signal. Mean ± SEM, student's t-test, $*p \leq 0.05$.

B) Slot blot for ADPr modification (left) and total RNA stain (right) following pulldown of biotinylated Y3 5' ysRNA oligonucleotide, or beads only control, after incubation with nuclear fractions from IR-treated (10 Gy, 10 mins, +IR) and untreated (-IR) cells, which had been subjected to siRNA-mediated knockdown of NSUN2 or DNMT2 or treated with a negative control siRNA (siNeg) for 48 h. Numbers represent signal intensity of ADPr in Y3 5' pulldown, normalised to corresponding total RNA signal. Quantified using ImageJ.

Supplementary Figure 11

A) Western blot showing levels of PARP1 and γH2AX, with GAPDH as a loading control, following IR treatment (10 Gy) at indicated time points (mins) of wild-type (WT) or YBX1 knockout (YBX1-/-) cells.

B) Ponceau stain for total protein levels corresponding to the Western blot shown in Figure 6B.

C) Western blot showing levels of PARP1 and γH2AX, with GAPDH as a loading control, following IR treatment (10 Gy) at indicated time points (mins) of wild-type (WT) or PARP1 knockout (PARP1-/-) cells.

D) Western blot showing levels of PARP1, with GAPDH as a loading control, following IR treatment (10 Gy) at indicated time points (mins) of Y3 or control (Luc) ASO-treated cells. Numbers represent fold change in normalised band intensity over control, quantified using ImageJ.

Name

Synthetic ysRNA oligonucleotides
Y1 3'
Y1 5'
Y3 5'
AlexaFluor488-Y1 3'
AlexaFluor488-Y1 5'
AlexaFluor488-Y3 5'
Biotin-Y1 5'
Biotin-Y3 5'
Biotin-Y1 3'

Antisense oligonucleotides
Luc ASO
Y1 ASO
Y3 ASO

siRNAs
siNeg
siNSUN2 3'-UTR
siDNMT2 siGENOME SMARTpool #M-006671-01

Probes
Y1
Y3

Antibodies
Anti-GAPDH Antibody Mouse / IgG2b
Anti-phospho-Histone H2A.X (Ser139) Antibody, clone JBW301
Phospho-Chk1 (Ser317)
CHK1 Polyclonal
HSP70 Polyclonal
TSG101 Polyclonal
Calnexin Polyclonal
Flotillin 1 Polyclonal
CD63 Polyclonal
Phospho-SMC1 (Ser966) Polyclonal
SMC1
Anti-Histone H3 (tri methyl K9)
Anti-YB-1 Rabbit Polyclonal
Purified Mouse Anti-Human PARP Clone 4C10-5 (RUO)
Anti-pan-ADP-ribose binding reagent
Phospho-gamma-H2AX (Ser139) Rabbit Monoclonal
5-Methylcytosine Rabbit Monoclonal Antibody (RM231)
Anti-Lamin B1 Rabbit Polyclonal
Histone-H3 Polyclonal
NSUN2 Mouse Monoclonal
DNMT2 [D-9] Mouse monoclonal

Sequence : (5' to 3')

UUUCCCCCCUUCUCACUACUGCACUUGACU
GGCUGGUCCGAAGGUAGUGAGUUAUCUCAA
GGCUGGUCCGAGUGCAGUGGUGUUUACAAC
5Alexa488N/UUUCCCCCCUUCUCACUACUGCACUUGACU
5Alexa488N/GGCUGGUCCGAAGGUAGUGAGUUAUCUCAA
5Alexa488N/GGCUGGUCCGAGUGCAGUGGUGUUUACAAC
5BiotinTEG/GGCUGGUCCGAAGGUAGUGAGUUAUCUCAA
5BiotinTEG/GGCUGGUCCGAGUGCAGUGGUGUUUACAAC
5BiotinTEG/UUUCCCCCCUUCUCACUACUGCACUUGACU

T*+T*T*T*+T*C*T*C*C*T*T*C*T*T*C*A*+G*A*+T*T
A*+T*T*G*+A*T*A*A*C*T*C*A*C*T*+A*C*+C*T
A*+G*T*T*+G*T*A*A*A*C*A*C*C*A*C*T*+G*C*+A*C

UGGUUUACAUGUCGACUAA
UAUUCCAAGGAUCCCAACCdTdT
GCGAUAUGCUCUUCUGUUA; CAGAAGAAAUUCACAGGAA; GAACGUUGAACCAAAUAUU; CAAAUUCAAGGCUAC[illegible]

TCACTACCTTCGGACCAGCC
CCACTGCACTCGGACCAGCC

Proteintech #60004-1-IG
Millipore #05-636
Cell Signalling Technologies #2344
Proteintech #25887-1-AP
Proteintech #10995-1-AP
Proteintech #14497-1-AP
Proteintech #10427-2-AP
Proteintech #15571-1-AP
Proteintech #25682-1-AP
Bethyl #A300-050A
Cell Signalling Technologies #4802
Abcam #ab8898
Abcam #ab12148
BD Biosciences #556494
Sigma #MABE1016
ThermoFisher #MA5-33062
ThermoFisher #MA5-24694
Abcam #ab16048
Proteintech #17168-1-AP
Proteintech #66580-1-Ig
Santa Cruz #sc-365001

GAUA
